# Supplementary material for: SNIP1 and PRC2 coordinate cell fates of neural progenitors during brain development
Source: Nat Commun. 2023 Aug 8;14:4754. doi: 10.1038/s41467-023-40487-4 (PMC10409800; doi:10.1038/s41467-023-40487-4)
Supplement: Supplementary file 1 — Supplementary Information [file 41467_2023_40487_MOESM1_ESM.pdf]

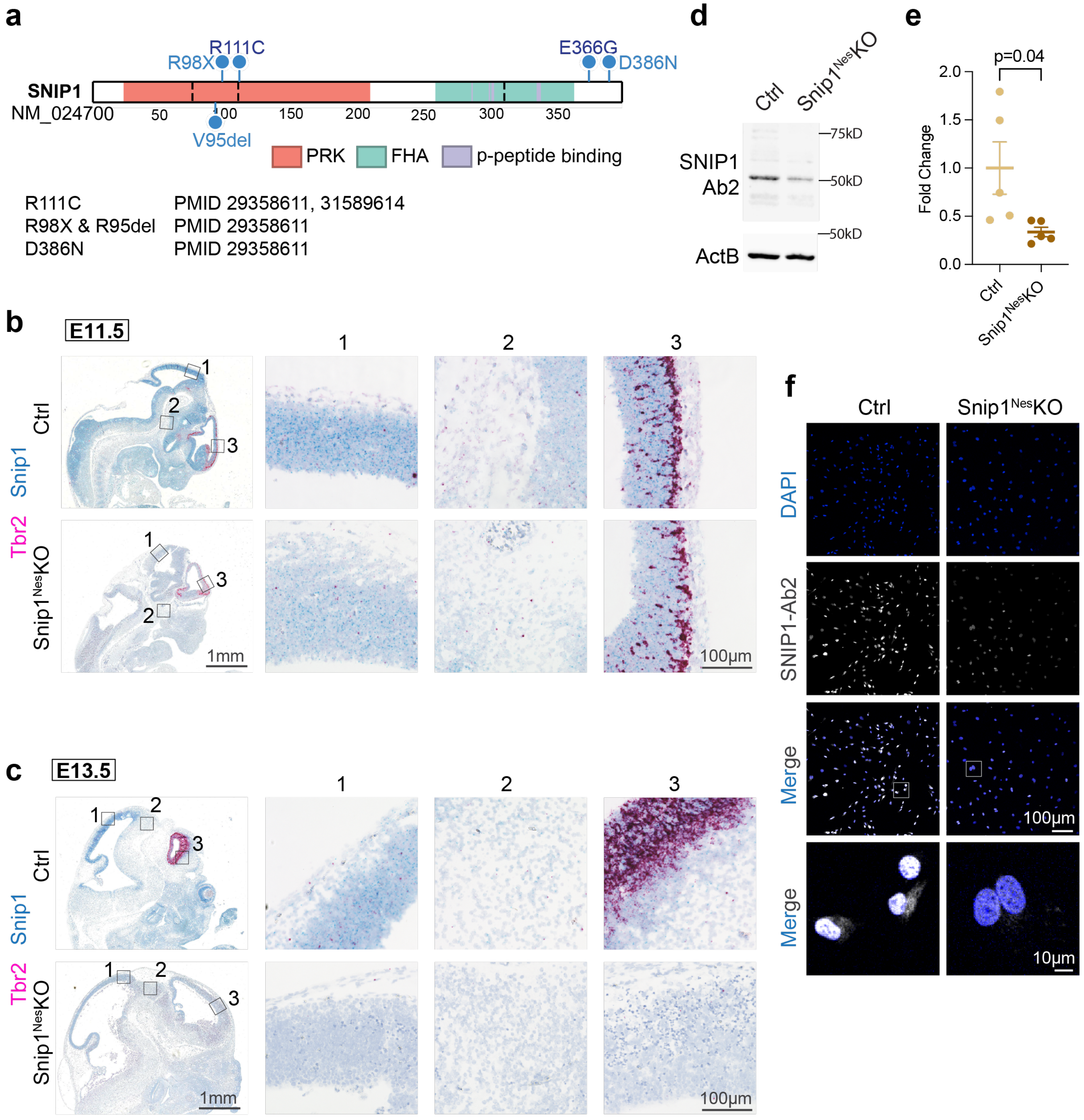

Supplementary Fig. 1 *Snip1* transcript is detected throughout the developing brain with strong expression in the neuroepithelia.

a Graphical summary of *SNIP1* variants that are associated with epilepsy and skull dysplasia. PMID reference of the source data is listed for the unpublished variants. The R111C variant passed statistical threshold, whereas the other 3 variants nearly passed statistical threshold.

b-c RNAscope in situ hybridization of control and *Snip1*^{Nes}-KO cryosections at (**b**) E11.5 and (**c**) E13.5. Three magnified representative regions are shown in (**1-3**). At E13.5, robust SNIP1 expression (in teal) was detected in the neuroepithelia of the control embryo, whereas it is reduced in the *Snip1*^{Nes}-KO embryo. Cells positive for the intermediate progenitor marker TBR2 (in red) were reduced in the *Snip1*^{Nes}-KO embryo. One pair of control and *Snip1*^{Nes}-KO was analyzed at each embryonic age. Bar, 1 mm (entire brain view) and 100 μm (magnified view).
d WB of control and *Snip1*^{Nes}-KO brains at E13.5. SNIP1-Ab2; anti-SNIP1 antibody from ThermoFisher.
e Quantification of WB immunoblotted with anti-SNIP1 antibodies. The band intensity of SNIP1 was normalized to that of ACTB. N=5 embryos per genotype. Data are presented as mean ± SEM, and two-tailed unpaired t test was performed for statistical significance.
f IF of SNIP1 (Ab2; ThermoFisher antibody) and DAPI in control and *Snip1*^{Nes}-KO cultured NPCs. Two replicates of IF showed similar results. Bar, 100 μm (entire view) and 10 μm (magnified view).
Source data are provided in a Source Data file (**d**, **e**).

Matsui_Supplementary Figure 2

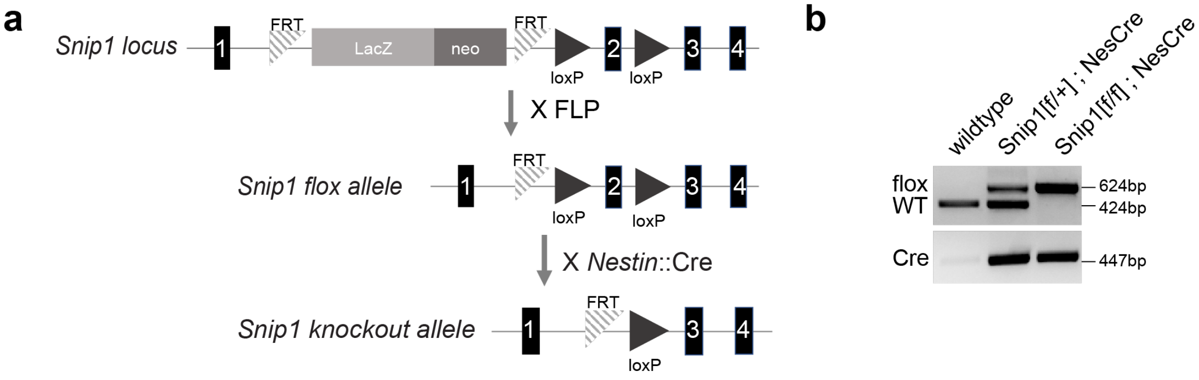

c

E13.5

Genotype	Exp.	Obs.	X^2	p-value
Snip1[+/+]; NesCre	9.5	12	-	-
Snip1[f/+]; NesCre	19	14	1.92	0.17
Snip1[f/f]; NesCre	9.5	12	0	>0.99
Total	38	38	2.63	0.27

d

weaning

Genotype	Exp.	Obs.	X^2	p-value
Snip1[+/+]; NesCre	15	38	-	-
Snip1[f/+]; NesCre	30	22	4.27	0.039
Snip1[f/f]; NesCre	15	0	38	<0.0001
Total	60	60	52.4	<0.0001

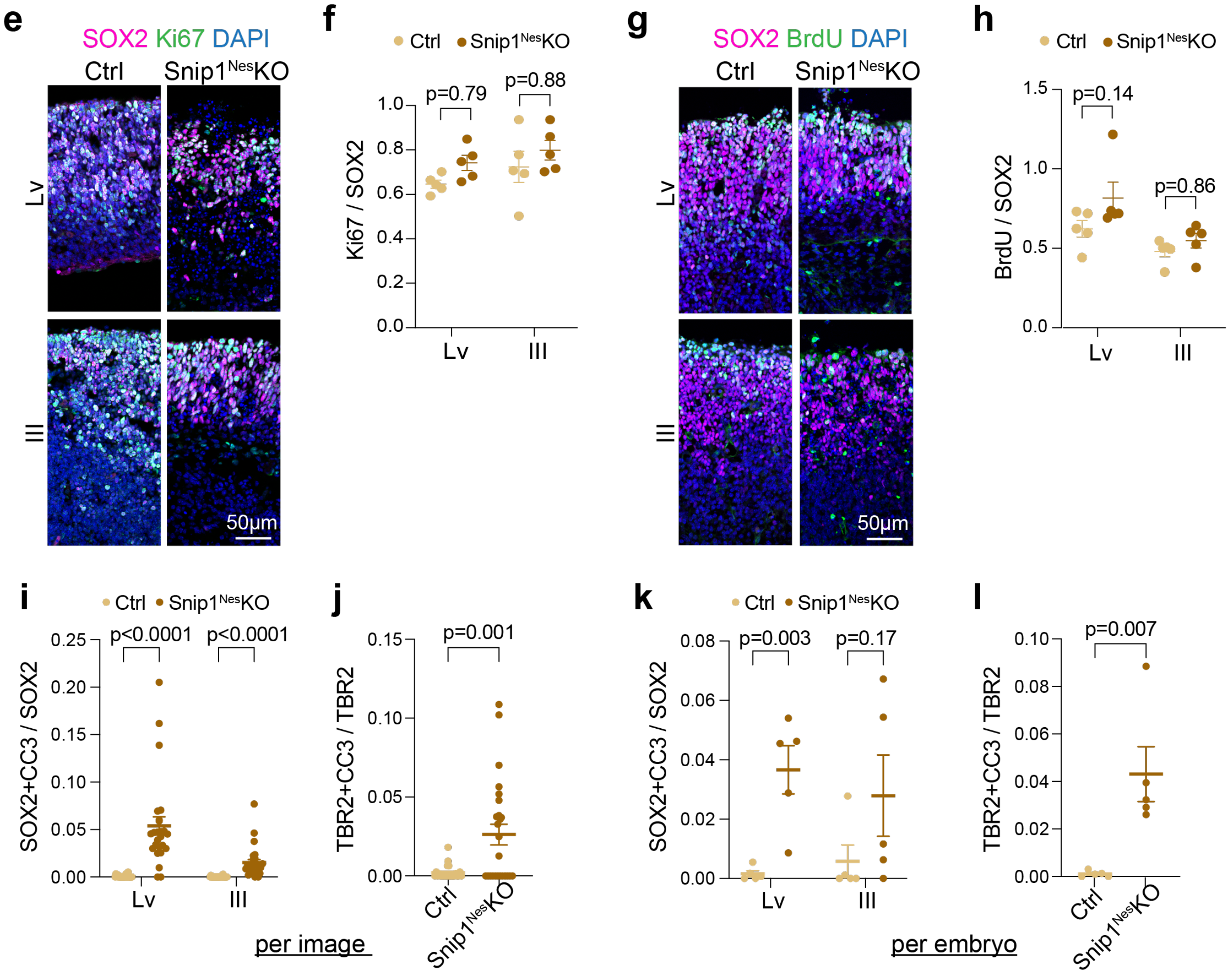

Supplementary Fig. 2 Mice with SNIP1-depleted NPCs are not viable by weaning age.

a Schematic representation of *Snip1* locus containing the LacZ cassette and loxP sites flanking exon 2. Excision of the LacZ cassette by flippase (FLP) generates *Snip1*[flox] allele. Subsequent excision of exon 2 by Cre recombinase driven by *Nestin* promoter (*Nes*::Cre) generates *Snip1* knockout allele.

b Genotyping PCR of wildtype allele, *Snip1*[flox] allele, and allele with Cre recombinase transgene. This genotyping PCR was performed for all of the embryos dissected for this paper (n>500).

c-d Viability of *Snip1*$[+/+]^{Nes}$, *Snip1*$[flox/+]^{Nes}$ and *Snip1*$[flox/flox]^{Nes}$ mice at (**c**) E13.5 and at (**d**) the weaning age (between 3 and 4 weeks after birth). Two-tailed chi-square test was performed to determine if the mice of three genotypes are viable at the Mendelian ratio. $Snip1^{Nes}$-KO embryos were obtained at the expected Mendelian frequency at E13.5; however, no $Snip1^{Nes}$-KO mice were obtained at the weaning age.

e, g IF of SOX2 and (**e**) Ki67 or (**g**) BrdU in DAPI-stained sagittal cryosections of the E13.5 brain. Germinal zones around lateral and 3rd ventricles were examined. Bar, 50 µm.

f, h Quantification of BrdU-positive, proliferating NPCs in control and $Snip1^{Nes}$-KO embryos at E13.5. The plot compares one representative sibling pair, and each data point represents one image. N=5 images per genotype. Data are presented as mean ± SEM, and two-way ANOVA was used for statistical analysis.

i-l Quantification of cell populations that are double-positive for SOX2 and CC3 (**i,k**) or TBR2 and CC3 (**j,l**). For (**i,j**), each data point represents one image with n=24 control and n=25 $Snip1^{Nes}$-KO. For (**k,l**), each data point represents one embryo with n=5 embryos per genotype. Data are presented as mean ± SEM, and two-tailed unpaired t-tests were used for statistical analysis.

Source data are provided in a Source Data file (**b**, **f**, **h-l**).

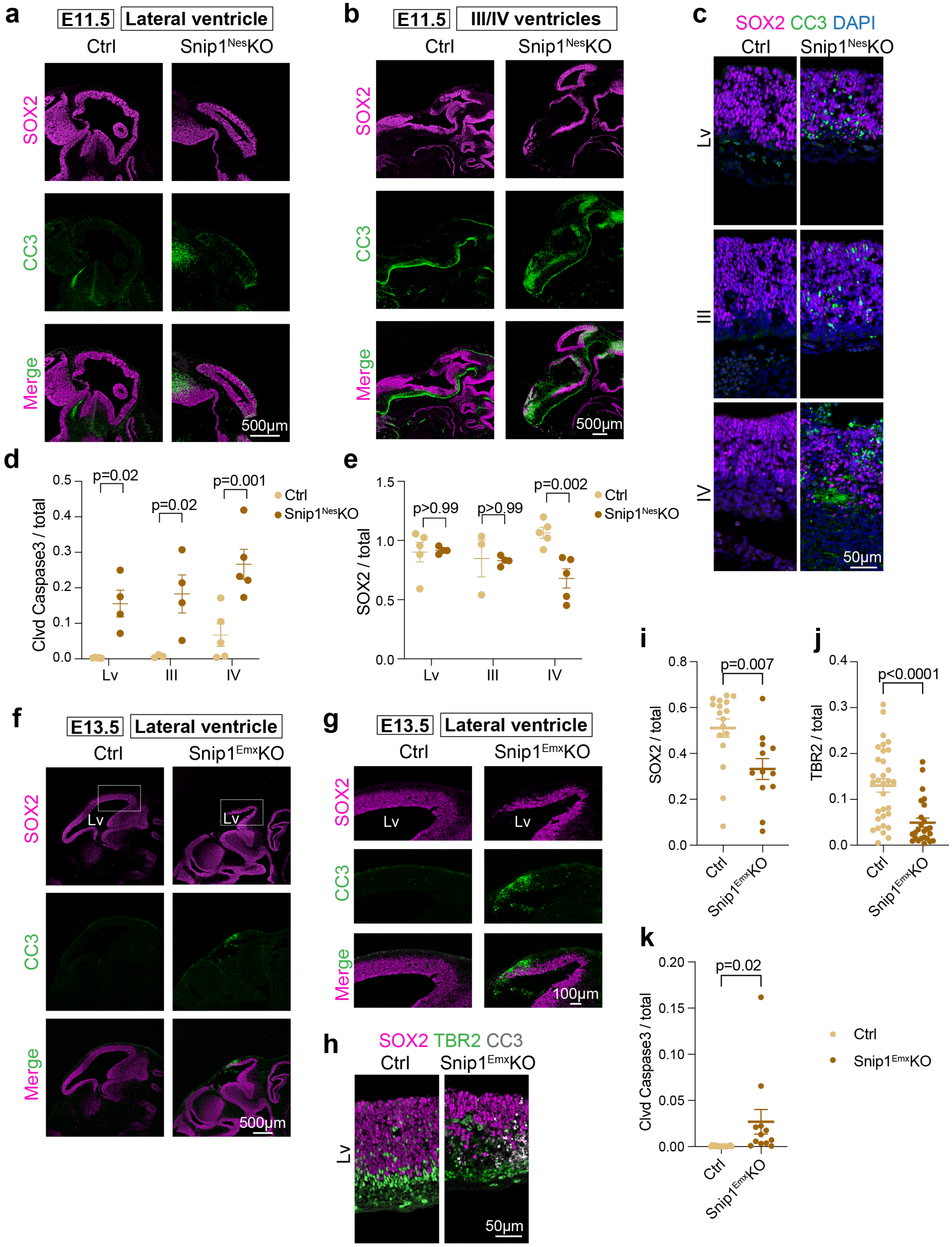
a
E11.5 Lateral ventricle
Ctrl
Snip1NesKO
SOX2
CC3
Merge
500µm
b
E11.5 III/IV ventricles
Ctrl
Snip1NesKO
SOX2
CC3
Merge
500µm
c
SOX2 CC3 DAPI
Ctrl
Snip1NesKO
Lv
III
IV
50µm
d
Clvd Caspase3 / total
p=0.02
p=0.02
p=0.001
Ctrl
Snip1NesKO
Lv
III
IV
e
SOX2 / total
p>0.99
p>0.99
p=0.002
Ctrl
Snip1NesKO
Lv
III
IV
f
E13.5 Lateral ventricle
Ctrl
Snip1EmxKO
SOX2
CC3
Merge
Lv
500µm
g
E13.5 Lateral ventricle
Ctrl
Snip1EmxKO
SOX2
CC3
Merge
Lv
100µm
h
SOX2 TBR2 CC3
Ctrl
Snip1EmxKO
Lv
50µm
i
SOX2 / total
p=0.007
Ctrl
Snip1EmxKO
j
TBR2 / total
p<0.0001
Ctrl
Snip1EmxKO
k
Clvd Caspase3 / total
p=0.02
Ctrl
Snip1EmxKO
Ctrl
Snip1EmxKO

Supplementary Fig. 3 Induction of apoptosis occurs in the *Snip1^{Nes}*-KO brains as early as E11.5.

a-b IF of SOX2 and CC3 in sagittal cryosections of the E11.5 brain. Germinal zones around (**a**) lateral ventricle and (**b**) 3rd/4th ventricles were examined. Bar, 500 μm.

c IF with a higher magnification staining against SOX2, CC3, and DAPI of the E11.5 brain. Bar, 50 μm.

d-e Quantification of CC3-positive and SOX2-positive cells in the neuroepithelial lining of the ventricles of control and *Snip1^{Nes}*-KO embryos at E11.5. DAPI staining was used to count the total number of cells. Each data point represents one image. For control, n=5 (Lv), 3 (III), and 5 (IV) images. For *Snip1^{Nes}*-KO, n=4 (Lv), 4 (III), and 5(IV) images. Data are presented as mean ± SEM, and two-way ANOVA was used for statistical analysis.

f-g IF of SOX2 and CC3 in sagittal cryosections of control and *Snip1^{Emx1}*-KO brains at E13.5. Germinal zones around lateral ventricle was examined. Bar, (**f**) 500 μm and (**g**) 100 μm.

h IF of SOX2 and CC3 overlayed with an intermediate progenitor marker TBR2 in control and *Snip1^{Emx1}*-KO brains at E13.5. Bar, 50 μm.

i-k Quantification of (**i**) SOX2-positive, (**j**) TBR2-positive, and (**k**) CC3-positive cells in the neuroepithelial lining of lateral ventricle of control and *Snip1^{Emx1}*-KO embryos at E13.5. DAPI staining was used to count the total number of cells. Each data point represents one image. For (**I,k**), n=17 (control) and n=12 (*Snip1^{Emx1}*-KO). For (**j**), n=32 (control) and n=25 (*Snip1^{Emx1}*-KO) images. Data are presented as mean ± SEM, and two-tailed unpaired t test was used for statistical analysis.

Source data are provided in a Source Data file (**d**, **e**, **i-k**).

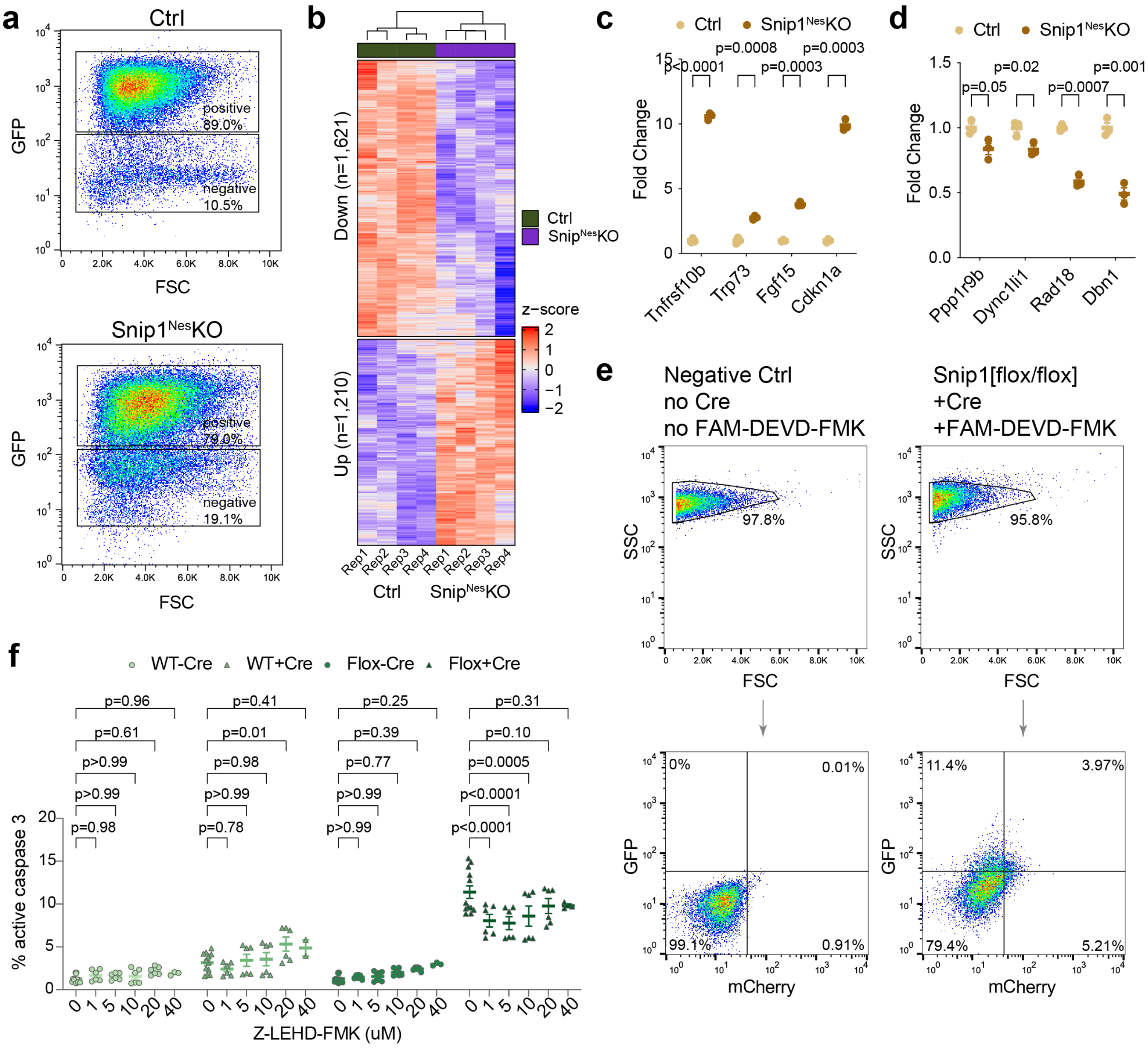

<u>Supplementary Fig. 4</u> <u>Differential gene expression between control and *Snip1^{Nes}*-KO NPCs.</u>

a FACS density plots of the control and *Snip1^{Nes}*-KO brain cells at E13.5. The proportion of the GFP-positive population is reduced in the *Snip1^{Nes}*-KO vs. control brains.

b Heatmap of differentially expressed genes (by the criterion of FDR<0.05) in *Snip1^{Nes}*-KO and control NPCs.

c-d Quantitative PCR of the representative genes that were (**c**) upregulated or (**d**) downregulated in the *Snip1^{Nes}*-KO NPCs versus control NPCs. The Cq values of each gene were normalized to those of a housekeeping gene *Actb*. For each gene, a fold change of *Snip1^{Nes}*-KO samples relative to control

samples was calculated. N=3 for each genotype. Data are presented as mean ± SEM, and two-tailed unpaired t-tests with Welch correction were used for statistical analysis.

e Example FACS density plots showing the sample gating strategies. FSC/SSC and mCherry/GFP plots of the negative control and *Snip1*[flox/flox] NPCs transduced with mCherry-Cre lentivirus are shown.

f The percentage of cells with active caspase 3 quantified by FACS. Caspase 9 inhibitor (Z-LEHD-FMK) was added at different concentrations along with mCherry-Cre lentivirus. The percentage of FAM-FLICA (active caspase 3)-positive population (out of total population) is shown. N=12 for DMSO control, n=6 for the 1, 5, 10, and 20μM samples, n=3 for the 40μM WT-Cre, Flox-Cre, and Flox+Cre samples, and n=2 for the 40μM WT+Cre sample. Data are presented as mean ± SEM, and two-way ANOVA was used for statistical analysis. The representative FACS plots are shown in **Supp Fig 14a**.

Source data are provided in a Source Data file (**c**, **d**, **f**).

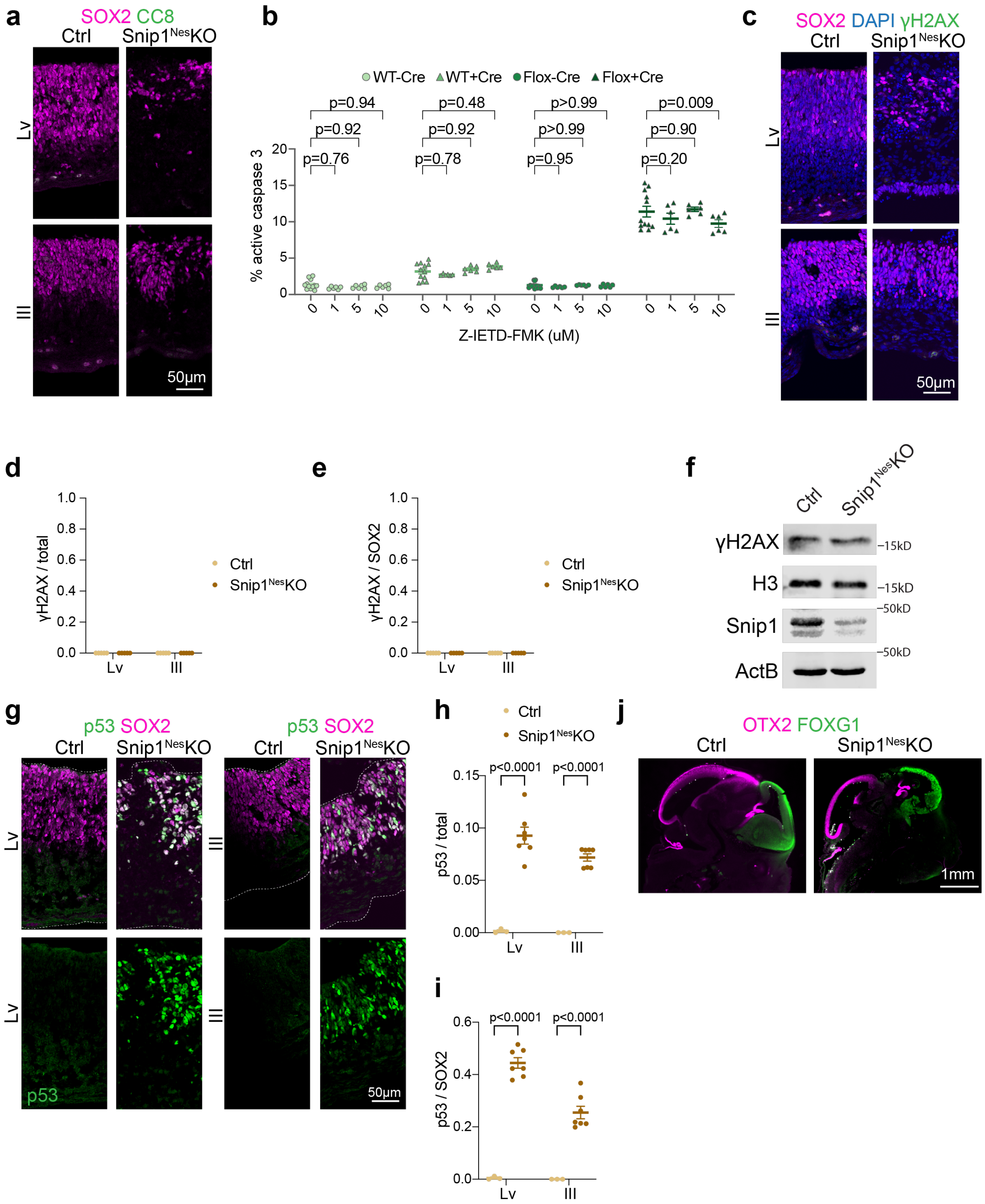

Supplementary Fig. 5 *Snip1*^{Nes}-KO-induced cell death does not involve DNA damage response.

a IF of cleaved caspase 8 (CC8) and SOX2 in sagittal cryosections of E13.5 brains. Bar, 50 µm.
b Quantification of cells with active caspase 3 quantified by FACS. SNIP1 was depleted in *Snip1*[flox/flox] NPCs by lentiviral mCherry-Cre while cells were treated with a caspase 8 inhibitor Z-IETD-FMK at different concentrations. The percentage of FAM-FLICA (active caspase 3)-positive population (out of total population) is shown. N=12 for DMSO control and n=6 for the rest of the samples. Data are presented as mean ± SEM, and two-way ANOVA was used for statistical analysis. The representative FACS plots are shown in **Supp Fig 14b**.
c IF of γH2AX and SOX2 in sagittal cryosections of E13.5 brains. Bar, 50 µm.
d-e Quantification of the population of γH2AX-positive cells relative to (**d**) the total number of cells by DAPI or (**e**) the number of SOX2-positive cells in the neuroepithelial lining of the ventricles of control and *Snip1*^{Nes}-KO embryos at E13.5. Each data point represents one image with n=5 per genotype. None of the observed cells were detected as γH2AX-positive in either control or *Snip1*^{Nes}-KO embryos.
f WB analysis of the nuclear extract from control and *Snip1*^{Nes}-KO brains at E13.5.
g IF of p53 and SOX2 in sagittal cryosections of E13.5 brains. Bar, 50 µm. Little p53 signal was detected in the control brain, whereas p53 was detected in a large proportion of the SOX2-positive cells in *Snip1*^{Nes}-KO brains.
h-i Quantification of the population of p53-positive cells relative to (**h**) the total number of cells by DAPI or (**i**) the number of SOX2-positive cells in the neuroepithelial lining of the ventricles of control and *Snip1*^{Nes}-KO embryos at E13.5. Each data point represents one image with n=3 for control and n=7 for *Snip1*^{Nes}-KO. Data are presented as mean ± SEM, and two-way ANOVA was used for statistical analysis.
j IF of FOXG1 (a forebrain marker) and OTX2 (a mid/hindbrain marker) in control and *Snip1*^{Nes}-KO brains at E13.5. Two replicates of IF showed similar results. Bar, 1 mm.
Source data are provided in a Source Data file (**b**, **e-f**, **h**, **i**).

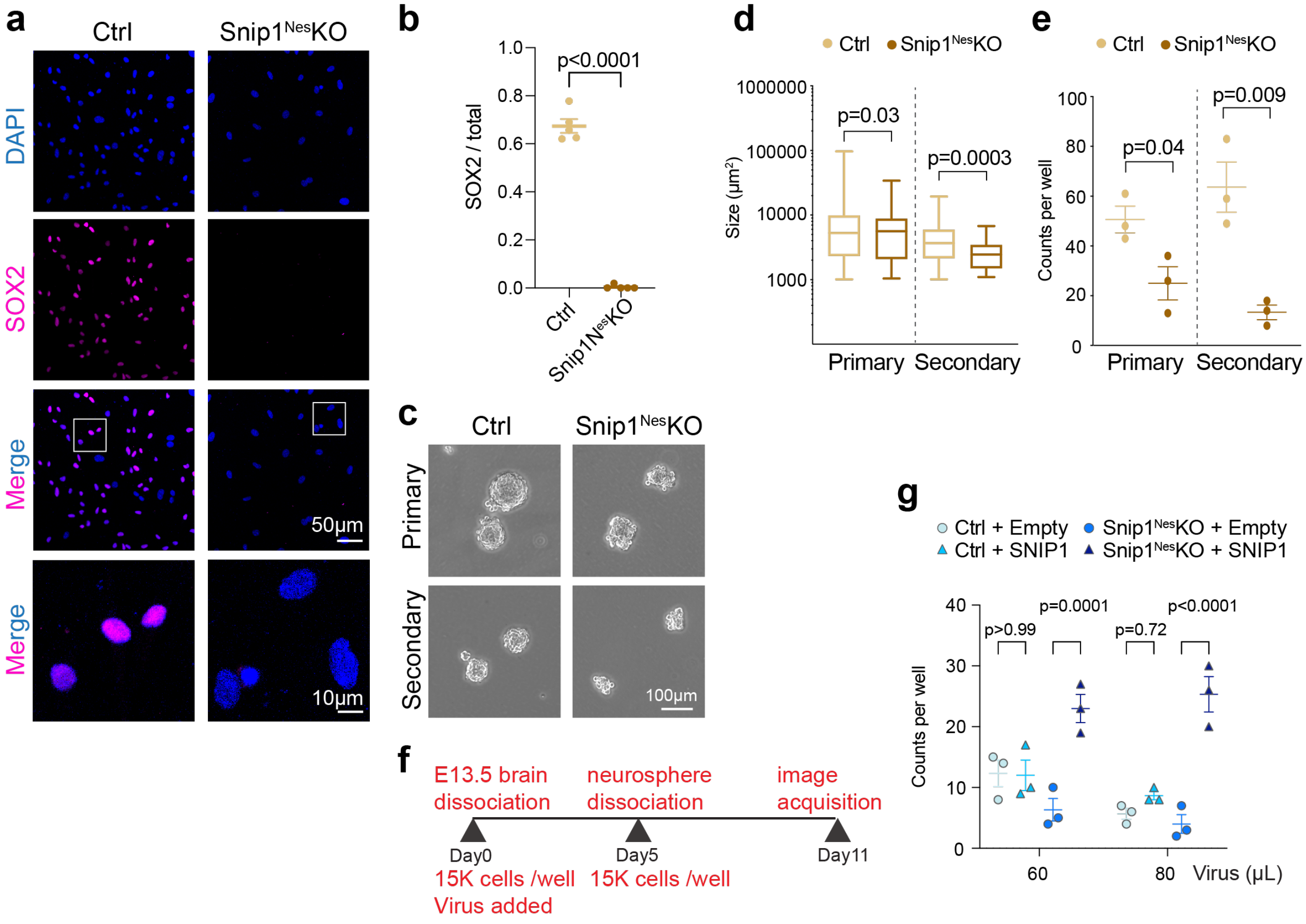

<u>**Supplementary Fig. 6** Effect of SNIP1 on NPC self-renewal.</u>

a IF of SOX2 and DAPI in control and *Snip1^{Nes}*-KO cultured NPCs. Bar, 50 μm (entire view) and 10 μm (magnified view).

b Quantification of SOX2-positive cells as represented in **Supp Fig 6a**. DAPI staining was used to quantify the total number of cells. Each data point represents one image with n=5 per genotype. Data are presented as mean ± SEM, and two-tailed t test was used for statistical analysis.

c Brightfield images of the sequential neurosphere assay of control and *Snip1^{Nes}*-KO NPCs. For primary neurosphere formation, 10,000 cells were seeded to each well of low-attachment 6-well plates. For secondary neurosphere formation, primary neurospheres were dissociated and 3,000 cells were seeded to each well. *Snip1^{Nes}*-KO neurospheres did not grow well and therefore, we failed to obtain the seeding number of 10,000 cells for the secondary neurosphere. All neurospheres were imaged after 5 days of culture. Bar, 100 μm.

d-e Quantification of the neurosphere size and counts per well. For (**d**), the number of neurospheres were n=152 primary control, n=75 primary *Snip1*^{Nes}-KO, n=191 secondary control, and n=40 secondary *Snip1*^{Nes}-KO. For (**e**), n=3 wells were counted. Data are presented as (**d**) box plots and (**e**) mean ± SEM. For (**d**), each box extends from the 25th to 75th percentiles with the central line showing the median. The whiskers indicate the smallest and the largest values. Two-tailed unpaired t-test was used for statistical analysis.
f Schematic of a neurosphere rescue experiment.
g Quantification of the counts of neurospheres per well. N=3 wells were counted. Data are presented as mean ± SEM, and two-way ANOVA was used for statistical analysis. Overexpressing human SNIP1 in *Snip1*^{Nes}-KO NPCs increased neurosphere formation.
Source data are provided in a Source Data file (**b**, **d**, **e**, **g**).

Matsui_Supplementary Figure 7

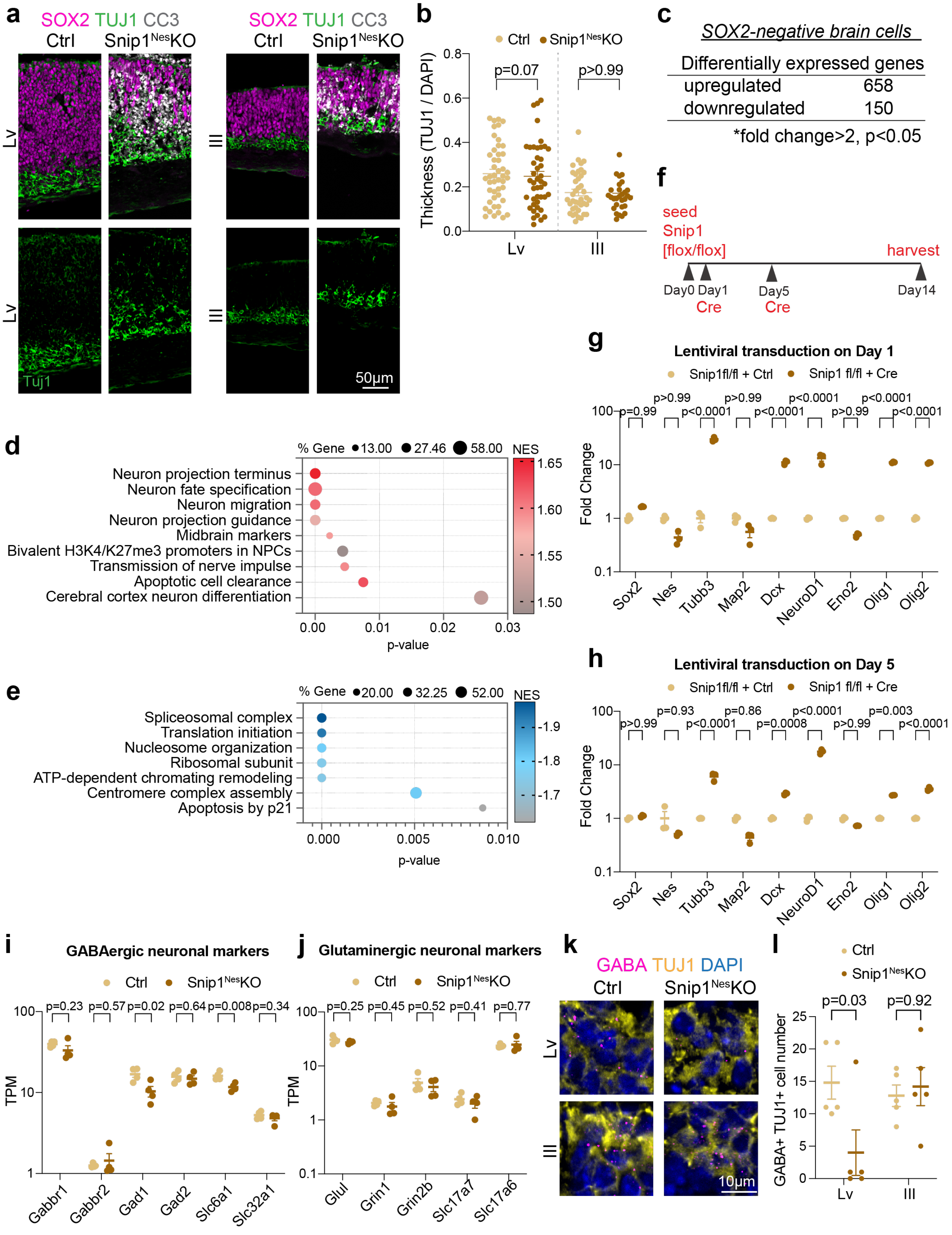

Supplementary Fig. 7 Effect of SNIP1 on NPC differentiation.

a IF of SOX2 and CC3 overlayed with a premature neuronal marker TUJ1 of the E13.5 brain. Bar, 50 µm.

b Thickness of the TUJ1-positive region relative to the entire cortical thickness. Each data point represents one image with n=46 for control (Lv), n=44 for $Snip1^{Nes}$-KO (Lv), n=40 for control (III), and n=29 for $Snip1^{Nes}$-KO (III). Data are presented as mean ± SEM, and two-way ANOVA was used for statistical analysis.

c The numbers of differentially expressed genes in the SOX2-negative cells of the control and $Snip1^{Nes}$-KO brains at E13.5. Two RNA-seq datasets each of control and $Snip1^{Nes}$-KO cells were analyzed. The numbers of genes that passed the cutoff of fold-change >2 and p <0.05 are shown.

d-e Bubble plots of the enriched gene sets in (**d**) upregulated genes and (**e**) downregulated genes in $Snip1^{Nes}$-KO vs. control SOX2-negative brain cells. Differentially expressed genes were first ranked by their fold-change and p-value before GSEA was performed. P-values were calculated by a right-sided permutation test with FDR adjustment.

f Schematic of in vitro neural differentiation assay. *Snip1*[flox/flox] NPCs were transduced with mCherry-Cre lentivirus on Day 1 or Day 5 and RNAs were harvested on Day 14.

g-h Quantitative PCR of neuronal and glial markers on Day 14. Cells were depleted of SNIP1 either on (**g**) Day 1 or (**h**) Day 5 during in vitro differentiation. N=3 for each genotype. Data are presented as mean ± SEM, and two-way ANOVA was used for statistical analysis.

i-j Transcript levels of (**i**) GABAergic neuronal markers and (**j**) glutaminergic neuronal markers in SOX2 -negative brain cells. TPM (transcript per million) of each gene is shown. N=4 embryos per genotype. Data are presented as mean ± SEM, and two-tailed unpaired t tests were used for statistical analysis.

k IF of a GABAergic neuronal marker GABA overlayed with TUJ1 and DAPI of the E13.5 brain. Bar, 10 µm.

l Quantification of GABA-positive cells that are also TUJ1-positive in the control and $Snip1^{Nes}$-KO brains at E13.5. Cells that are positive for both GABA and TUJ1 in the area of 212.55 μm^2 were counted. Each data point represents one 212.55 μm^2 image with n=5 per genotype. Data are presented as mean ± SEM, and two-way ANOVA was used for statistical analysis.

Source data are provided in a Source Data file (**b**, **d**, **e**, **g-j**, **l**).

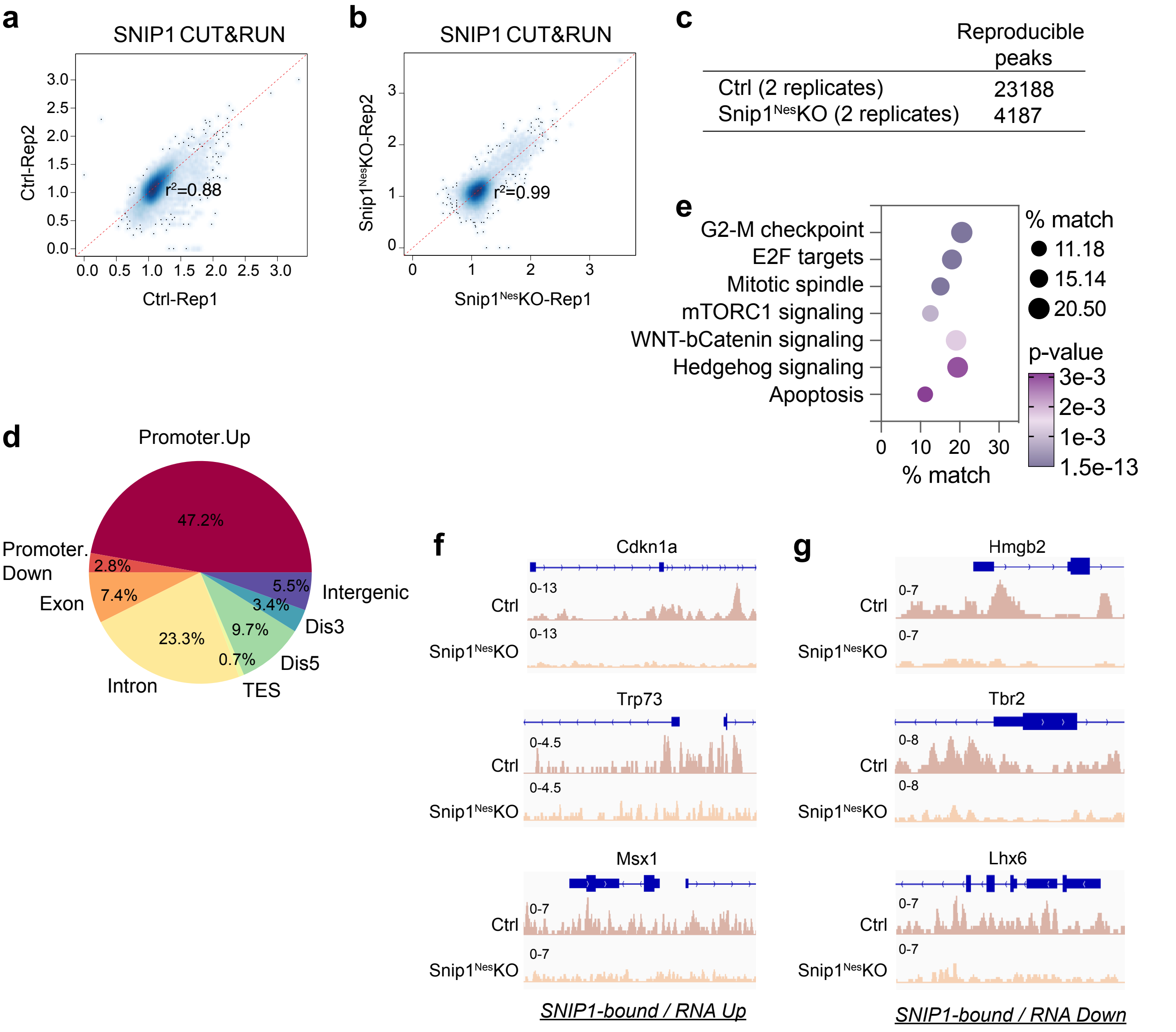

Supplementary Fig. 8 Profiling of SNIP1 binding to chromatin by CUT&RUN.

a-b Pearson correlation plot showing the variance between replicates for SNIP1 CUT&RUN.

c The number of reproducible peaks bound by SNIP1 in control and *Snip1^{Nes}*-KO NPCs. Peaks were called by merging both SICER and mac2 peaks with the cutoff of FDR <0.05 and FDR <0.5. A peak is considered reproducible if it is detected with at least FDR <0.05 in one replicate and no more than FDR >0.5 in the others (see Methods).

d Proportions of SNIP1 binding to genomic features. Fifty percent of SNIP1 binding was detected in promoter regions.

e Gene ontology of the 1,093 genes downregulated in *Snip1*^{Nes}-KO and bound by SNIP1 only in WT. Genes were searched against gene ontology resources using Enrichr[56]. P-values were calculated by a right-sided Fisher's exact test adjusted with the Benjamini & Hochberg method. Source data are provided in a Source Data file.

f-g SNIP1 CUT&RUN tracks visualized by Integrative Genomics Viewer (IGV) at (**f**) upregulated genes and (**g**) downregulated genes. *Cdkn1a*, Chr17: 29,090,888 - 29,095,850. *Trp73,* Chr4: 154,132,565 - 154,143,373. *Msx1*, Chr5: 37,818,429 - 37,828,924. *Nes*, Chr3: 87,970,718 - 87,974,908. *Tbr2*, Chr9: 118,476,575 - 118,480,298. *Lhx6*, Chr2: 36,101,041 - 36,106,574.

Matsui_Supplementary Figure 9

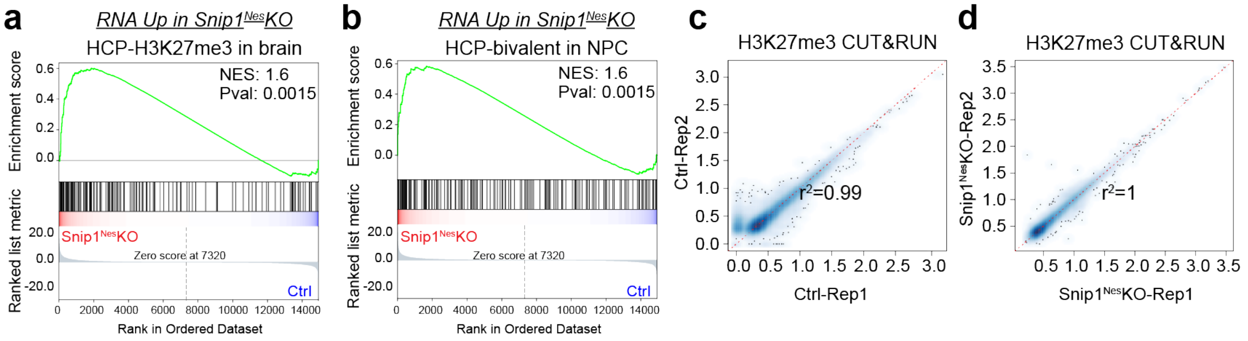

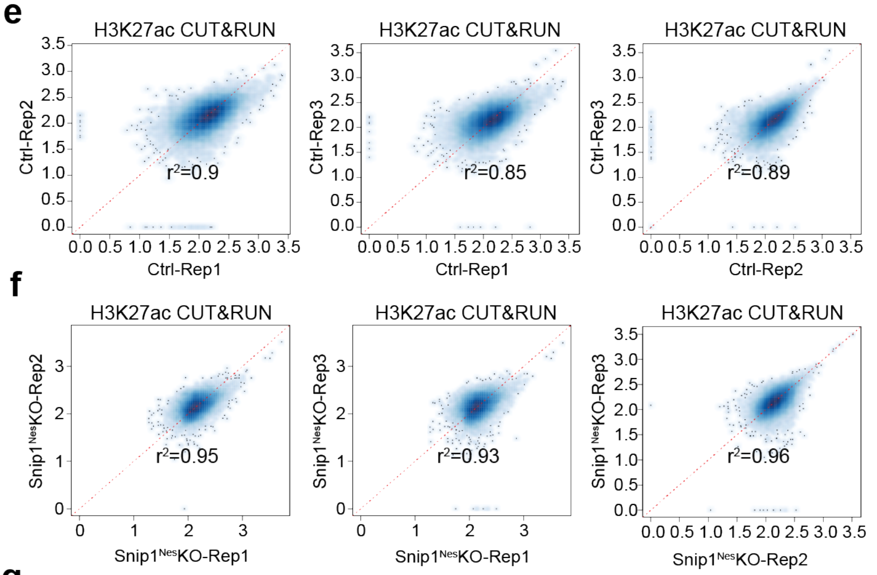

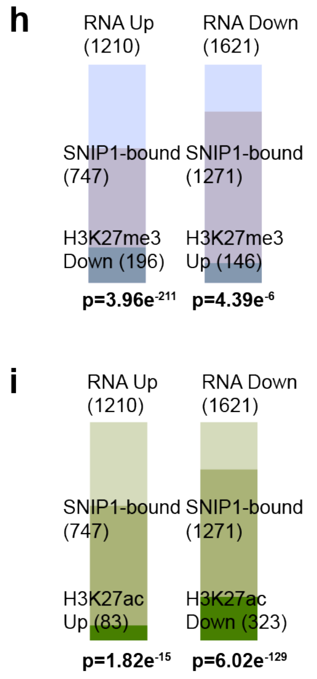

g

		Reproducible peaks
H3K27me3	Ctrl (2 replicates)	38442
	Snip1^{Nes}KO (2 replicates)	33402
H3K27ac	Ctrl (3 replicates)	13610
	Snip1^{Nes}KO (3 replicates)	18738

j

Category	Genes
Upregulated intrinsic apoptosis (44)	Eda2r, Phlda3, Cdkn1a, Perp, Aen, Creb3l1, Msx1, Epha2, Tnfrsf1a, Ddias, Bbc3, Trp73, Nkx3-1, Mmp9, Creb3, Nme5, Hic1, Rrp8, Rps27l, Tmem109, Syvn1, Wfs1, Pias4, Hmox1, Bax, Snai1, Cd44, Selenos, Nfe2l2, Ybx3, Tmem117, Bcap31, Cebpb, Mybbp1a, Rad9a, Slc9a3r1, Lrrk2, Mdm2, Flcn, Ppp1r13b, Sfn, Tnfrsf1b, Clu, Bak1
Snip1-bound (37)	Eda2r, Phlda3, Cdkn1a, Aen, Creb3l1, Msx1, Epha2, Tnfrsf1a, Ddias, Bbc3, Trp73, Mmp9, Creb3, Nme5, Hic1, Rrp8, Rps27l, Tmem109, Syvn1, Wfs1, Pias4, Hmox1, Bax, Selenos, Nfe2l2, Ybx3, Tmem117, Bcap31, Mybbp1a, Rad9a, Slc9a3r1, Lrrk2, Mdm2, Flcn, Ppp1r13b, Clu, Bak1
H3K27me3 down (9)	Cdkn1a , Aen, Msx1, Trp73, Epha2, Hic1, Tmem117, Ppp1r13b, Sfn
H3K27ac up (5)	Phlda3, Aen, Bbc3, Wfs1, Nfe2l2

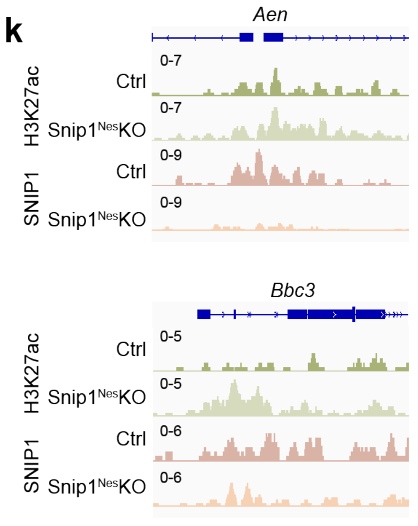

Supplementary Fig. 9 Profiling of H3K27me3 and H3K27ac in control and *Snip1*^{Nes}-KO NPCs.

a-b Representatives of GSEA of upregulated genes in *Snip1*^{Nes}-KO NPCs. Upregulated genes were enriched in (**a**) high-CpG-density promoters with H3K27me3 in the embryonic murine brain and (**b**) high-CpG-density promoters with bivalent (H3K27me3 and H3K4me3) marks in mouse NPCs. Differentially expressed genes were first ranked by their fold-change and p-value before GSEA was performed. P-values were calculated by a right-sided permutation test with FDR adjustment.

c-f Pearson correlation plots between replicates in control and *Snip1*^{Nes}-KO samples for (**c-d**) H3K27me3 and (**e-f**) H3K27ac CUT&RUN.

g The number of reproducible peaks enriched with H3K27me3 or H3K27ac in control and *Snip1*^{Nes}-KO NPCs. Peaks were called by merging both SICER and macs2 peaks with the cutoff of FDR <0.05 and FDR <0.5. A peak is considered reproducible if it is detected with at least FDR <0.05 in one replicate and no more than FDR >0.5 in the others (see Methods).

h-i Bar charts displaying the proportions of differentially expressed genes that were occupied by SNIP1 and/or exhibited changes in (**h**) H3K27me3 or (**i**) H3K27ac levels. Right-sided hypergeometric test was performed for statistical analysis.

j Lists of upregulated intrinsic apoptosis genes in *Snip1*^{Nes}-KO vs. control NPCs under three different categories corresponding to **Fig 3g**.

k H3K27ac and SNIP1 CUT&RUN tracks visualized by Integrative Genomics Viewer (IGV) at upregulated intrinsic apoptosis genes. H3K27ac levels increased at the presented loci in *Snip1*^{Nes}-KO. *Aen*, Chr7:78,894,346 - 78,897,964. *Bbc3,* Chr7:16,307,660 - 16,311,277.

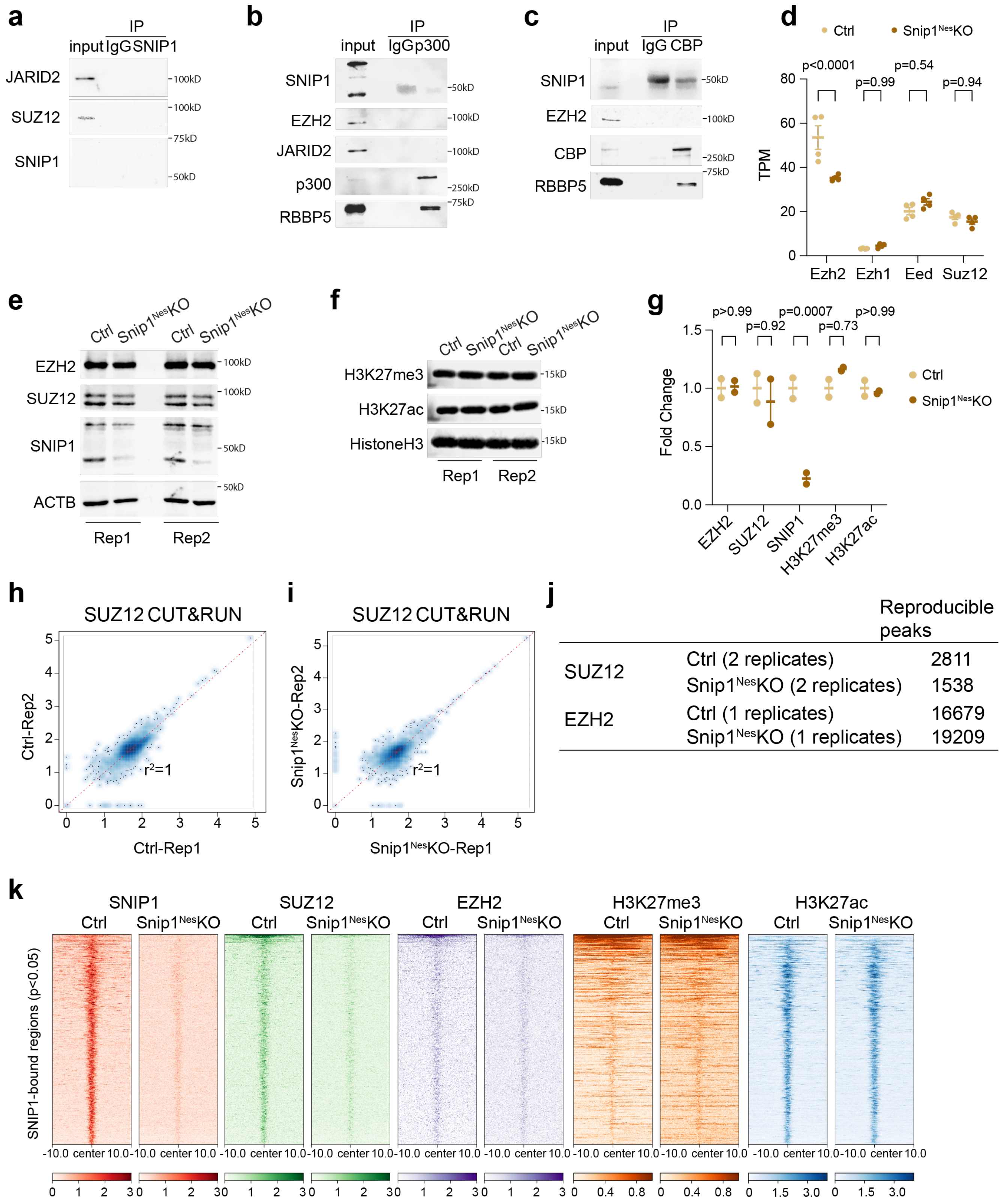
a
IP
input IgG SNIP1
JARID2
SUZ12
SNIP1
100kD
100kD
75kD
50kD
b
IP
input IgG p300
SNIP1
EZH2
JARID2
p300
RBBP5
50kD
100kD
100kD
250kD
75kD
c
IP
input IgG CBP
SNIP1
EZH2
CBP
RBBP5
50kD
100kD
250kD
75kD
d
Ctrl
Snip1NesKO
p<0.0001
p=0.99
p=0.54
p=0.94
TPM
Ezh2 Ezh1 Eed Suz12
e
Ctrl Snip1NesKO Ctrl Snip1NesKO
EZH2
SUZ12
SNIP1
ACTB
100kD
100kD
50kD
50kD
Rep1 Rep2
f
Ctrl Snip1NesKO Ctrl Snip1NesKO
H3K27me3
H3K27ac
HistoneH3
15kD
15kD
15kD
Rep1 Rep2
g
p>0.99
p=0.92
p=0.0007
p=0.73
p>0.99
Fold Change
Ctrl
Snip1NesKO
EZH2 SUZ12 SNIP1 H3K27me3 H3K27ac
h
SUZ12 CUT&RUN
Ctrl-Rep2
Ctrl-Rep1
r2=1
i
SUZ12 CUT&RUN
Snip1NesKO-Rep2
Snip1NesKO-Rep1
r2=1
j
Reproducible peaks
SUZ12 Ctrl (2 replicates) 2811
Snip1NesKO (2 replicates) 1538
EZH2 Ctrl (1 replicates) 16679
Snip1NesKO (1 replicates) 19209
k
SNIP1 SUZ12 EZH2 H3K27me3 H3K27ac
Ctrl Snip1NesKO
SNIP1-bound regions (p<0.05)
-10.0 center 10.0

Supplementary Fig. 10 SNIP1 depletion reduces chromatin occupancy of PRC2 without changing their protein expression.

a Co-immunoprecipitation followed by WB to examine the interaction between SNIP1 and PRC2 subunits in SNIP1-depleted NPCs. *Snip1*[flox/flox] NPCs were transduced with mCherry-Cre lentivirus for depleting SNIP1. Experiments were repeated at least twice and showed similar results.

b-c Co-immunoprecipitation followed by WB to examine the interaction between SNIP1 and p300/CBP. (**b**) p300 or (**c**) CBP was immunoprecipitated in the NPC nuclear extract. Anti-IgG antibody was used as the control for IP and RBBP5 was a positive control for p300 and CBP interactions. Experiments were repeated at least twice and showed similar results.

d Transcript levels of PRC2 subunits in SOX2-positive NPCs. TPM (transcript per million) of each gene is shown. N=4 per genotype. Data are presented as mean ± SEM, and two-way ANOVA was used for statistical analysis.

e-f WB of control and *Snip1*^{Nes}-KO brains at E13.5.

g Quantification of WB blots shown in **Supp Fig 10e**–**f**. The band intensities of SUZ12, EZH2, and SNIP1 were normalized to that of ACTB and the band intensities of H3K27me3 and H3K27ac were normalized to that of Histone H3. N=2 per genotype. Data are presented as mean ± SEM, and two-way ANOVA was used for statistical analysis.

h-i Pearson correlation plots between replicates in (**h**) control and (**i**) *Snip1*^{Nes}-KO samples for SUZ12 CUT&RUN.

j The number of reproducible peaks enriched with SUZ12 or EZH2 in control and *Snip1*^{Nes}-KO NPCs. Peaks were called by merging both SICER and macs2 peaks with the cutoff of FDR <0.05 and FDR < 0.5. A peak is considered reproducible if it is detected with at least FDR <0.05 in one replicate and no more than FDR >0.5 in the others (see Methods).

k Heatmaps aligning peaks of SUZ12, EZH2, H3K27me3, or H3K27ac at the SNIP1 targets. SNIP1 peaks that were reduced with p <0.05 in *Snip1*^{Nes}-KO vs. control were considered as true SNIP1 targets. A dark color indicates high intensity and a light color indicates low intensity.

Source data are provided in a Source Data file (**a**–**g**).

Matsui_Supplementary Figure 11

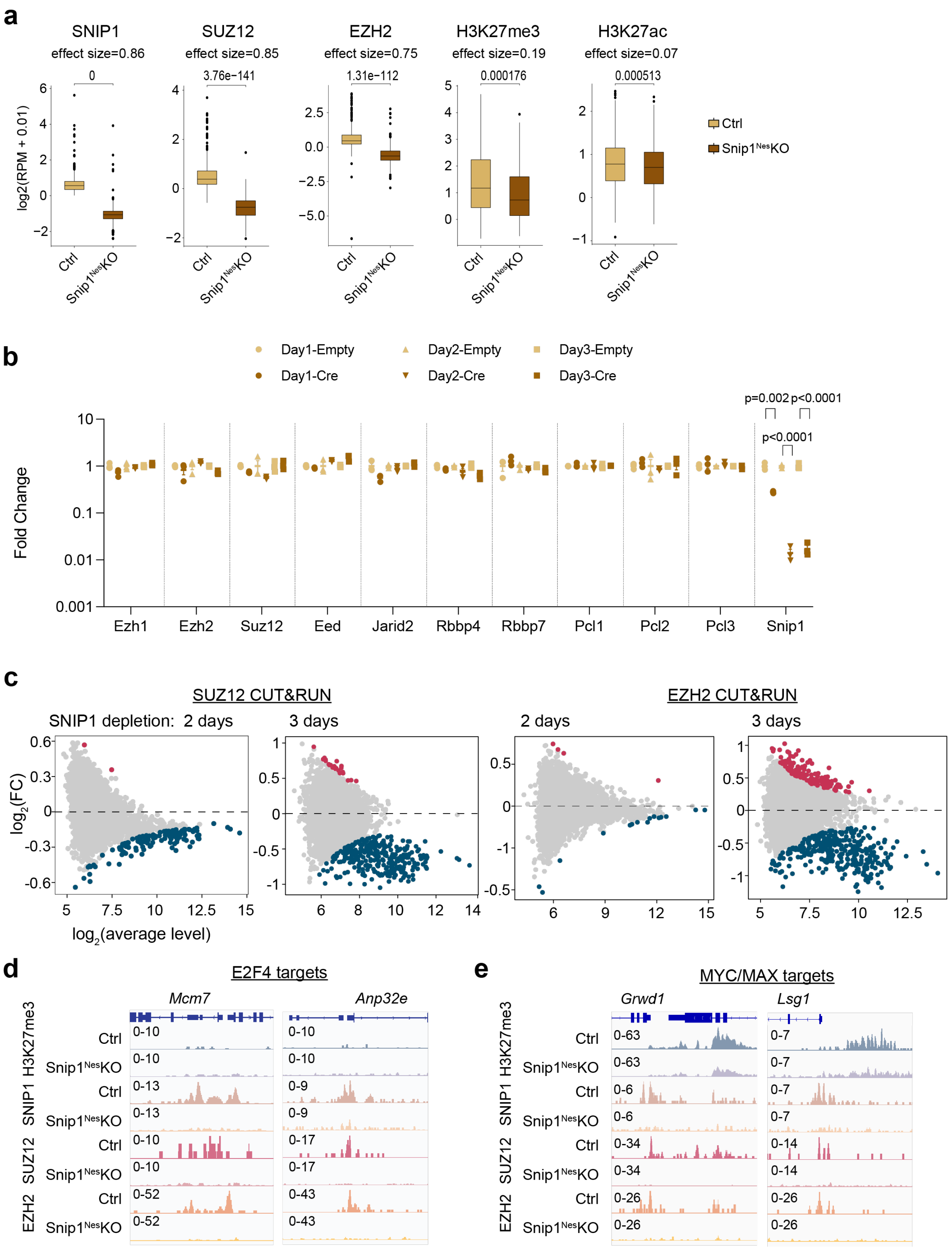

Supplementary Fig. 11 Profiling of PRC2 subunits on chromatin by CUT&RUN.

a Box plots comparing the binding intensity of SNIP1, PRC2, and H3K27me3/ac at the SNIP1 targets in *Snip1*^{Nes}-KO vs. control NPCs. For each boxplot, the black central line shows the median, the box limits indicate the interquartile range (25th-75th percentile), the whiskers indicate the 1.5 interquartile range excluding outliers, which are shown as individual dots. P-values were calculated by a right-sided Kruskal-Wallis test adjusted with the Benjamini & Hochberg method. The effect size was calculated as the Z statistic divided by the square root of the sample size ($Z/\sqrt{N}$).

b Transcript levels of PRC2 complex components in *Snip1*[flox/flox] NPCs after 1-3 days of treatment with lentiviral empty control. Normalization was to Actb and values at Day 1. N=3 per sample. Data are presented as mean ± SEM, and two-way ANOVA were used for statistical analysis. Source data are provided in a Source Data file.

c MA plot of SUZ12 and EZH2 binding regions in control vs. SNIP1-depleted NPCs. SNIP1 were depleted for either 2 or 3 days before CUT&RUN was performed.

d-e H3K27me3, SNIP1, and PRC2 CUT&RUN tracks visualized by Integrative Genomics Viewer (IGV) at the reported targets of (**d**) E2F4 (*Mcm7* and *Anp32e*) and (**e**) MYC/MAX (*Grwd1* and *Lsg1*). *Mcm7*, Chr5: 138,169,085 - 138,173,262. *Anp32e*, Chr3: 95,925,524 - 95,933,911. *Grwd1*, Chr7: 45,829,003 - 45,836,327. *Lsg1*, Chr16: 30,584,062 - 30,593,010.

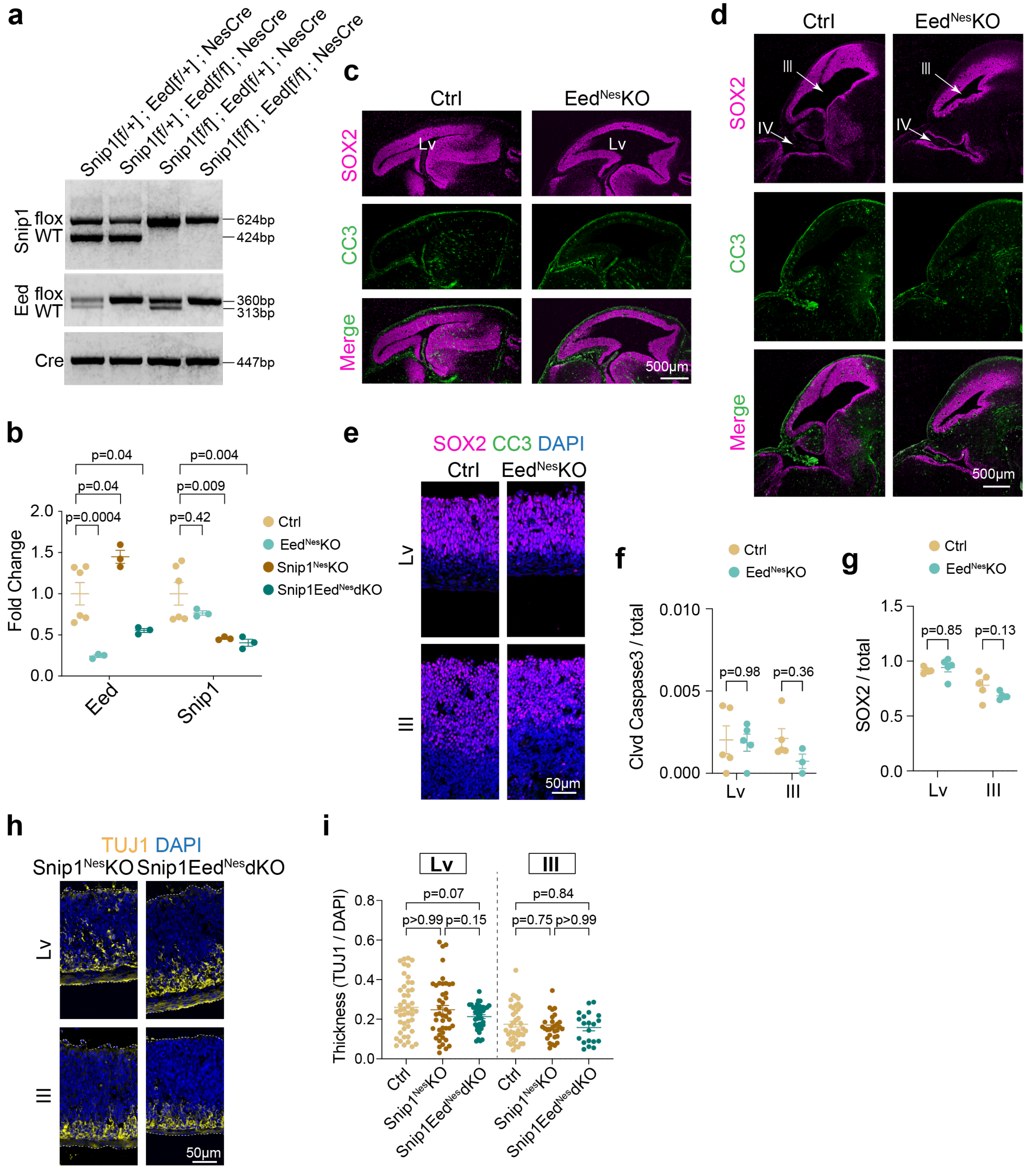

Supplementary Fig. 12 Characterization of *Eed*^{Nes}-KO and *Snip1*^{Nes}-*Eed*^{Nes}-dKO.

a Genotyping PCR of *Snip1*-flox allele, *Eed*-flox allele, and allele with *Nes*::Cre recombinase.
b Quantitative PCR of *Eed* and *Snip1* transcripts in the NPCs from E13.5 brains. The Cq values of each gene were normalized to that of a housekeeping gene *Gapdh*. For each gene, a fold change relative to the control brains was calculated. N=6 for control and n=3 for the other three genotypes. Data are presented as mean ± SEM, and two-way ANOVA was used for statistical analysis.
c-d IF analysis of SOX2 and CC3 in E13.5 brain. Bar, 50 μm. Germinal zones around (**c**) lateral ventricle (Lv) and (**d**) 3rd /4th ventricles were examined. Bar, 500 μm.
e IF analysis of SOX2 and CC3 in E13.5 brain. Bar, 50 μm.
f-g Quantification of CC3-positive and SOX2-positive cells in the neuroepithelial lining of the ventricles of control and *Eed*^{Nes}-KO embryos at E13.5. DAPI staining was used to quantify the total number of cells. Each data point represents one image with n=5 per genotype. Data are presented as mean ± SEM, and two-way ANOVA was used for statistical analysis.
h IF of TUJ1 in the E13.5 brain. Bar, 50 μm.
i Thickness of the TUJ1-positive region relative to the cortex. Each data point represents one image. For lateral ventricle, n=46 for control, n=44 for *Snip1*^{Nes}-KO, and n=42 for *Snip1*^{Nes}-*Eed*^{Nes}-dKO. For the third ventricle, n=40 for control, n=29 for *Snip1*^{Nes}-KO, and n=20 *Snip1*^{Nes}-*Eed*^{Nes}-dKO. Data are presented as mean ± SEM, and two-way ANOVA was used for statistical analysis.
Source data are provided in a Source Data file (**a**, **b**, **f**, **g**, **i**).

a

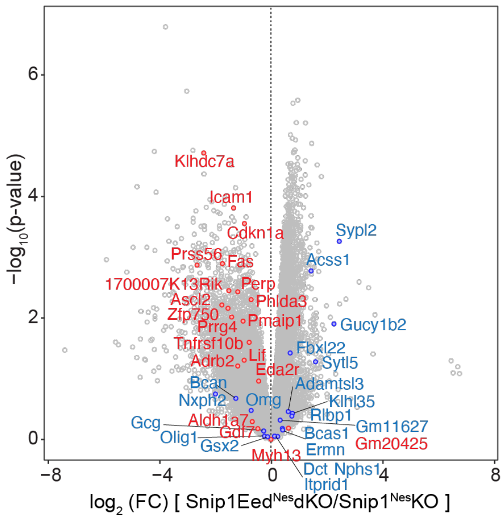

Differentially expressed genes	
upregulated	184
downregulated	994

*fold change>2, p<0.05

b

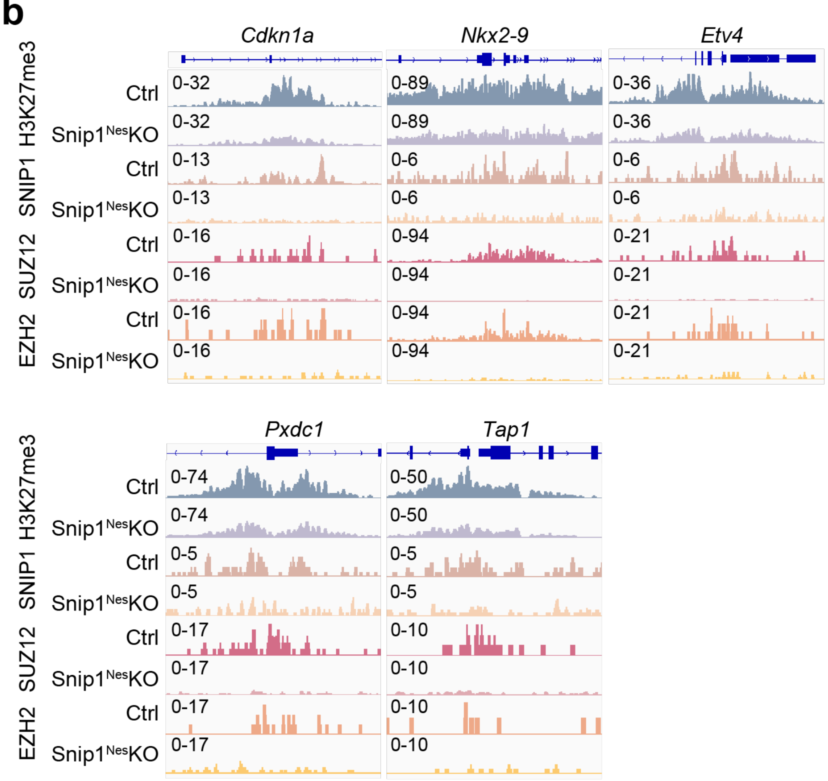

c

Gene	log_2(FC)	p-value	FDR
Cdkn1a	-0.95	2.79e-4	0.037
Ppp1r13b	-0.72	5.98e-3	0.091
Trp73	-1.59	8.49e-3	0.104
Hic1	-0.81	0.097	0.318
Msx1	-0.84	0.234	0.507
Tmem117	0.39	0.361	0.631
Sfn	-0.35	0.418	0.679
Aen	-0.15	0.465	0.715
Epha2	-0.04	0.889	0.940

*log_2 (FC) [Snip1Eed^{Nes}dKO/Snip1^{Nes}KO]

Supplementary Fig. 13 Differential gene expression of *Eed^{Nes}*-KO and *Snip1^{Nes}*-*Eed^{Nes}*-dKO.

a Volcano plot and the numbers of differentially expressed genes in *Snip1^{Nes}*-*Eed^{Nes}*-dKO vs. *Snip1^{Nes}*-KO. Genes that passed the cutoff of fold-change >2 and p <0.05 were counted. P-values were calculated by two-sided Voom-limma t test adjusted with the Benjamini & Hochberg method.

b H3K27me3, SNIP1, and PRC2 CUT&RUN tracks visualized by Integrative Genomics Viewer (IGV) at five of the 32 genes that were upregulated genes in *Snip1^{Nes}*-KO vs. control and downregulated genes in *Snip1^{Nes}*-*Eed^{Nes}*-dKO vs. *Snip1^{Nes}*-KO (corresponding to **Fig 6m**). *Cdkn1a*, chr17:29,090,577 -

29,097,359. *Nkx2-9*, chr12:56,606,409 - 56,618,370. *Etv4*, chr11:101,780,249 - 101,789,412. *Pxdc1*, chr13:34,648,803 - 34,656,057. *Tap1*, chr17:34,185,061 - 34,190,873.

c List of upregulated intrinsic apoptosis genes with reduced H3K27me3 in *Snip1*^{Nes}-KO NPCs corresponding to **Supp Fig 9j**. Fold change, p-value, and FDR of each gene comparing *Snip1*^{Nes}-*Eed*^{Nes}-dKO vs. *Snip1*^{Nes}-KO are shown.

a Fig 2h; Sup Fig 4f

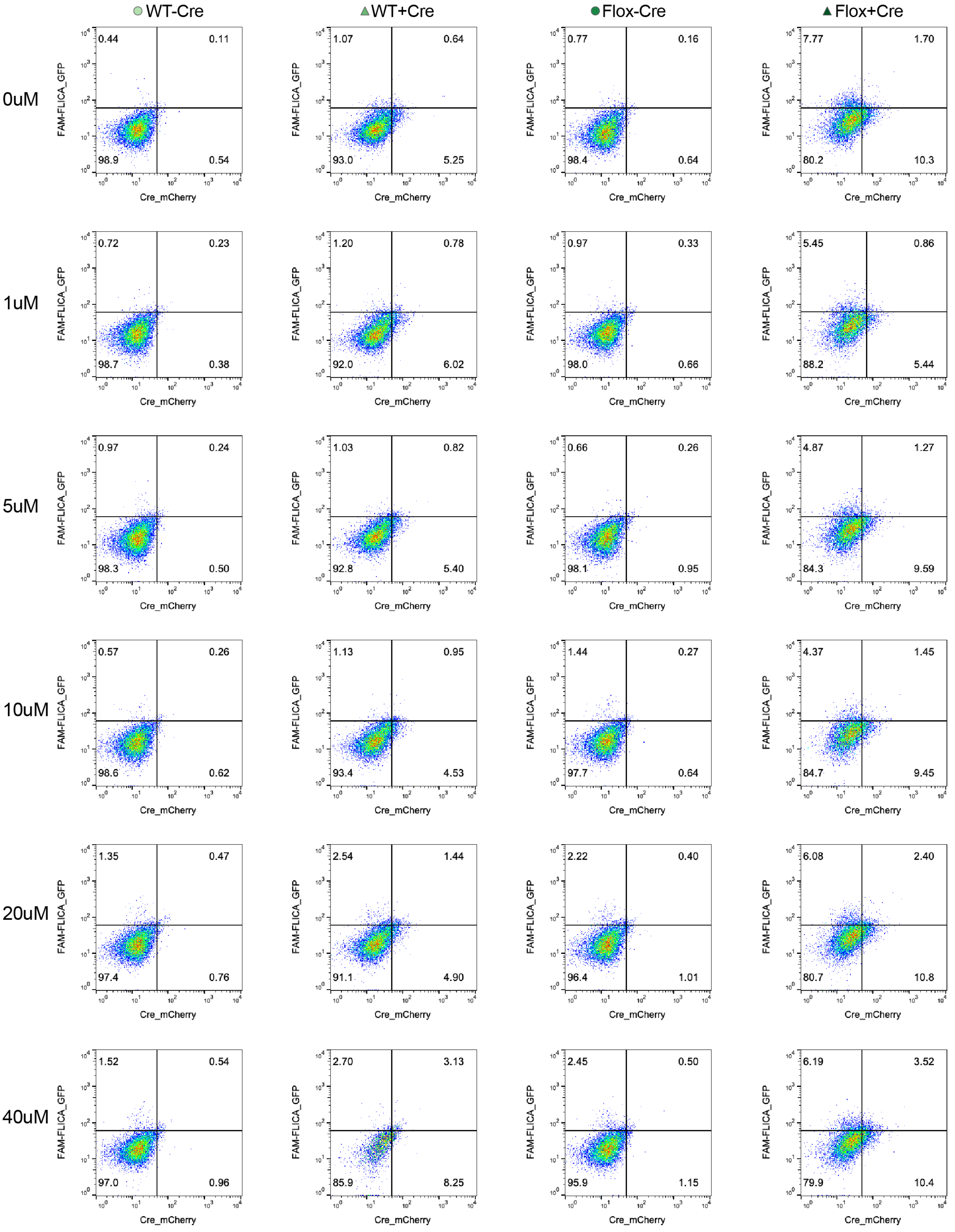

b **Sup Fig 5b**

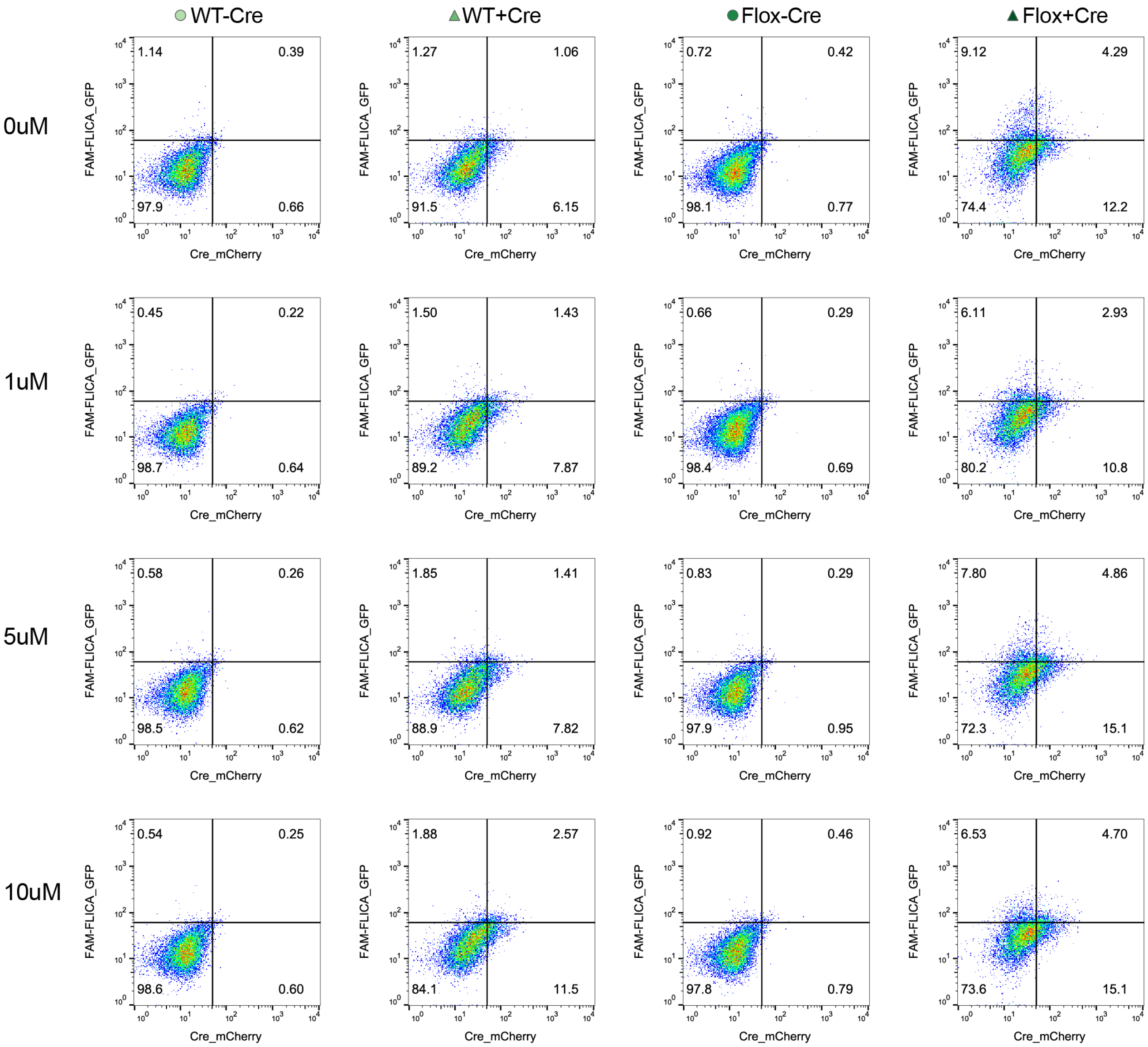
WT-Cre
WT+Cre
Flox-Cre
Flox+Cre
0uM
1uM
5uM
10uM
FAM-FLICA_GFP
Cre_mCherry
1.14
0.39
97.9
0.66
1.27
1.06
91.5
6.15
0.72
0.42
98.1
0.77
9.12
4.29
74.4
12.2
0.45
0.22
98.7
0.64
1.50
1.43
89.2
7.87
0.66
0.29
98.4
0.69
6.11
2.93
80.2
10.8
0.58
0.26
98.5
0.62
1.85
1.41
88.9
7.82
0.83
0.29
97.9
0.95
7.80
4.86
72.3
15.1
0.54
0.25
98.6
0.60
1.88
2.57
84.1
11.5
0.92
0.46
97.8
0.79
6.53
4.70
73.6
15.1

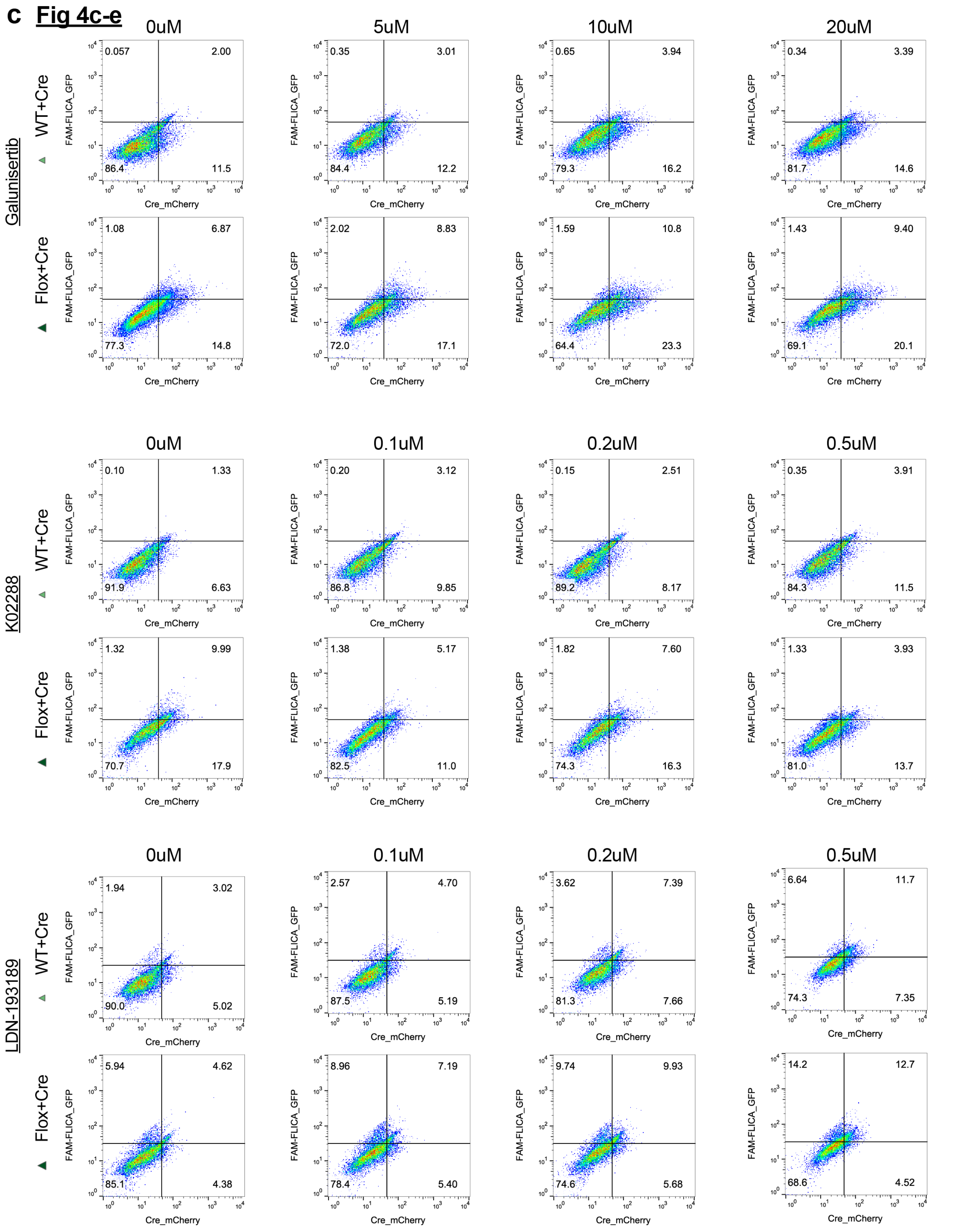
c Fig 4c-e
Galunisertib
0uM
5uM
10uM
20uM
WT+Cre
Flox+Cre
0.057 2.00 86.4 11.5
0.35 3.01 84.4 12.2
0.65 3.94 79.3 16.2
0.34 3.39 81.7 14.6
1.08 6.87 77.3 14.8
2.02 8.83 72.0 17.1
1.59 10.8 64.4 23.3
1.43 9.40 69.1 20.1
K02288
0uM
0.1uM
0.2uM
0.5uM
0.10 1.33 91.9 6.63
0.20 3.12 86.8 9.85
0.15 2.51 89.2 8.17
0.35 3.91 84.3 11.5
1.32 9.99 70.7 17.9
1.38 5.17 82.5 11.0
1.82 7.60 74.3 16.3
1.33 3.93 81.0 13.7
LDN-193189
0uM
0.1uM
0.2uM
0.5uM
1.94 3.02 90.0 5.02
2.57 4.70 87.5 5.19
3.62 7.39 81.3 7.66
6.64 11.7 74.3 7.35
5.94 4.62 85.1 4.38
8.96 7.19 78.4 5.40
9.74 9.93 74.6 5.68
14.2 12.7 68.6 4.52
FAM-FLICA_GFP
Cre_mCherry

d Fig 4f-h

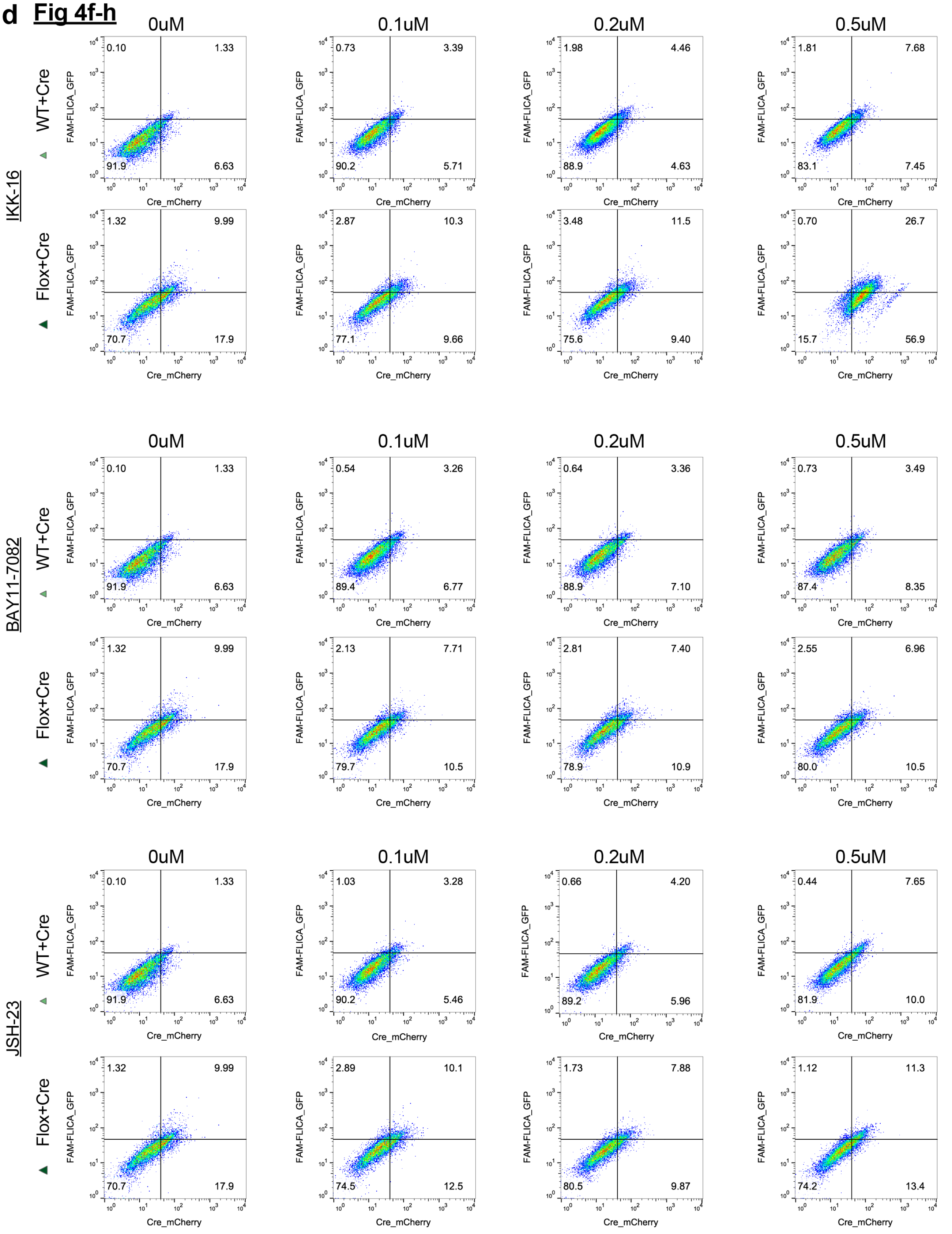
IKK-16
BAY11-7082
JSH-23
WT+Cre
Flox+Cre
0uM
0.1uM
0.2uM
0.5uM
FAM-FLICA_GFP
Cre_mCherry
0.10 1.33 91.9 6.63
0.73 3.39 90.2 5.71
1.98 4.46 88.9 4.63
1.81 7.68 83.1 7.45
1.32 9.99 70.7 17.9
2.87 10.3 77.1 9.66
3.48 11.5 75.6 9.40
0.70 26.7 15.7 56.9
0.10 1.33 91.9 6.63
0.54 3.26 89.4 6.77
0.64 3.36 88.9 7.10
0.73 3.49 87.4 8.35
1.32 9.99 70.7 17.9
2.13 7.71 79.7 10.5
2.81 7.40 78.9 10.9
2.55 6.96 80.0 10.5
0.10 1.33 91.9 6.63
1.03 3.28 90.2 5.46
0.66 4.20 89.2 5.96
0.44 7.65 81.9 10.0
1.32 9.99 70.7 17.9
2.89 10.1 74.5 12.5
1.73 7.88 80.5 9.87
1.12 11.3 74.2 13.4

<u>Supplementary Fig. 14</u> Representative FACS plots.

a Plots for Fig 2h and Supp Fig 4f.

b Plots for Supp Fig5b.

c Plots for Fig 4c-e.

d Plots for Fig 4f-h.

Supplementary Table 1. List of antibodies.

Target	Vendor	Catalog #	Assay (dilution)
Normal Rabbit IgG	RD Systems	AB-105-C	IP (4µg)<br>CUT&RUN (0.5-1µg)
Normal Goat IgG	RD Systems	AB-108-C	IP (4µg)
JARID2	Novus Biological	NB100-2214	IP (4µg)<br>WB (1:1000)
EZH2	Active Motif	39934	WB (1:1000)
EZH2	Active Motif	39076	IP (4µg)<br>WB (1:1000)<br>CUT&RUN (1µg)
SUZ12	Cell Signaling Technology	3737	WB (1:1000)<br>CUT&RUN (1µg)
SUZ12	Active Motif	39057	WB (1:1000)<br>CUT&RUN (1µg)
SNIP1	ThermoFisher	29412	IP (4µg)<br>IF (1:50)<br>WB (1:1000)<br>CUT&RUN (1µg)
SNIP1	ProteinTech	14950-I-AP	IP (4µg)<br>WB (1:1000)
RBBP5	Bethyl Laboratories	A300-109A	WB (1:1000)
p300	RD Systems	AF3789	IP (4µg)<br>WB (1:1000)
Cbp	GeneTex	GTX101249	IP (4µg)<br>WB (1:1000)
Drosophila H2Av (spike-in control)	Active Motif	61686	CUT&RUN (0.25ug)
p53	Leica Biosystems	NCL-L-p53-CM5p	IF (1:100)
Histone H3	Rockland	100-401-E81	WB (1:2000)
H3K27me3	Millipore	07-449	CUT&RUN (0.5µg)
H3K27ac	Abcam	ab4729	CUT&RUN (0.5µg)
γH2AX	Cell Signaling Technology	9718	IF (1:100)

γH2AX	Millipore	05-636	WB (1:1000)
β-Actin	Sigma-Aldrich	A1978	WB (1:2000)
SOX2	Santa Cruz Biotechnology	sc-17320	IF (1:150)
TBR2	ThermoFisher	14-4875-80	IF (1:200)
INSM1-Alexa Fluor® 488	Santa Cruz Biotechnology	sc-271408 AF488	IF (1:50)
TUJ1	Sigma-Aldrich	T8660	IF (1:300)
MAP2	Abcam	ab5392	IF (1:5000)
GABA	Sigma-Aldrich	A2052	IF (1:1000)
cleaved caspase 3 (Asp175)	Cell Signaling Technology	9661	IF (1:200)
cleaved caspase 8 (Asp387)	Cell Signaling Technology	8592	IF (1:200)
cleaved caspase 9 (Asp353)	Cell Signaling Technology	9509	IF (1:200)
Ki-67	Cell Signaling Technology	9129	IF (1:100)
BrdU	Santa Cruz Biotechnology	32323	IF (1:100)
FOXG1	Abcam	ab196868	IF (1:200)
OTX2	R&D Systems	AF1979	IF (1:150)
IRDye®-conjugated secondary	LI-COR	926-32213<br>926-68072	WB (1:17000)
Clean-Blot™ IP detection reagent	ThermoFisher	21230	WB (1:3000)
Alexa Fluor®-conjugated secondary	Jackson ImmunoResearch<br><br><br>ThermoFisher	712-545-153<br>703-545-155<br>705-605-147<br>A-31572<br>A-32766<br>A-32773	IF (1:500)

Supplementary Table 2. Genotype primers and conditions.

Line	Forward 5'->3'	Reverse 5'->3'	Anneal	Product Size (bp)
***Snip1*-tm1a**	GATGGAGCAGCATTGTAGGC	GAACTTCGGAATAGGAACTTCG	60 °C	234
***Snip1*-flox**	GATGGAGCAGCATTGTAGGC	CTTCTTGGCTGGGGACCTTT	60 °C	424(WT)<br>624(flox)
***Eed*-flox**	GGGACGTGCTGACATTTTCT	CTTGGGTGGTTTGGCTAAGA	52 °C	313(WT)<br>~360(flox)
***Actin*-FLPe**	ACTCCGTTAGGCCCTTCATT	GCATCATGTGCTGCTGAACT	56 °C	874
***Nestin*-Cre**	ATGCCCAAGAAGAAGAGGAAGGT	GAAATCAGTGCGTTCGAACGCTAGA	56 °C	447
***Emx1*-Cre**	ATGCCCAAGAAGAAGAGGAAGGT	GAAATCAGTGCGTTCGAACGCTAGA	56 °C	447
***Sox2*-eGFP**	CGTAAACGGCCACAAGTTCA	CTCAGGTAGTGGTTGTCGGG	56 °C	543

Supplementary Table 3. Primers for RT-qPCR.

	Gene	Forward 5'->3'	Reverse 5'->3'
Supp Fig 4c	Tnfrsf10b	ACCGACAGACATCTAGCACG	CGTTTACCGGAACCAGCAAC
	Trp73	GATGGCCCAGACCTCTTCTT	CTGGGGGAGGTCAAAGTAGG
	Fgf15	CTCCAACTGCTTCCTCCGAA	CGTCCTTGATGGCAATCGTC
	Cdkn1a	AATTGGAGTCAGGCGCAGAT	GAACAGGTCGGACATCACCA
Supp Fig 4d	Ppp1r9b	GATCCATTTCAGCACCGCAC	GCTCATATTCCGCAGAGGCT
	Dync1li1	ATGGGCAAGTGTGGTGAGAG	TCTTCTCCTGGCTCCACGTA
	Rad18	ACAGTGAATATCGTAAAAAGCACCA	CAGCTTGTGAAACTCTGCCA
	Dbn1	TCAGACAGCAGGAACGAGTG	TATGAAAGGGCAGTACGGACG
Supp Fig 7g,h	Sox2	ACAGCATGTCCTACTCGCAG	ATGCTGATCATGTCCCGGAG
	Nes	GTCTCAGGACAGTGCTGAGCCTTC	TCCCCTGAGGACCAGGAGTCTC
	Tubb3	TCTGGCGCCTTTGGACACCTATT	TTCTCACACTCTTTCCGCACGACA
	Map2	TGTGCTGTGTGCTCCAAGTT	GCTGGTGGTATGTTCTGGCT

	Dcx	CCTCTTTCTTCTCTTTTATTTGCCTTA	GGAACCACAGCAACTTTTCCAA
	NeuroD1	AGCTCCCACGTCTTCCACGTC	GGCTTTCAAAGAAGGGCTCCAG
	Eno2	CTCCCGCTGATCCTTCCCGATACA	ATGCCGACGTTGGCTGTGAACTTG
	Olig1	CTCGCCCAGGTGTTTTGTTG	TATAAGCCTGCGCTACGACG
	Olig2	TGGAGAGATGCGTTCGTTCC	GTGCTCTGCGTCTCGTCTAA
Supp Fig 11b, 12b	Ezh1	TCAACACTTCCCGCTGCAT	TGTCGAAGCCGCATATACTCA
	Ezh2	GTGACCACAGGATAGGCATCT	CAAGGGATTTCCATTTCTCG
	Suz12	GCATCAAAAGCTTGTCTGCAC	AGCAGGACTTCCAGGGTAAC
	Eed	AAGTTGAGCAGCGACGAGAA	ATTTGGCGTATTTGTGGGCG
	Jarid2	GGTCTGCTCAGGACTTACGG	TCTCGGATCTTTTTGGCACT
	Rbbp4	CTCCTGCAACGCACGAC	TTCTTCCACTGCGTCGTCAA
	Rbbp7	TAGAAGATGTGGAAGGGTGGG	TTCTTTGGTTGCTCATGTGGC
	Pcl1	AGGTGGGGTTAGCTACCTGT	CTAGGTACTGCACAGAGCCG
	Pcl2	GCTTGAAAATACACACTGGTGC	CAAAAGGAAATGGAAGCAAAGTTCT
	Pcl3	CCACCAGTCCTAACAAGGGG	TATAGCCCGTCTGTCCACCT
	Snip1	TCGGGAAGGAACTTTGAGGT	TCTTGGTCCGTGGTGACTTG
	Alt. Snip1	CCGGTCCCCAGCCAAG	TCCTCACGTTCCTGCTTCAC
	Gapdh	TCCCACTCTTCCACCTTCGATGC	GGGTCTGGGATGGAAATTGTGAGG
	βActin	TCCTCCTGAGCGCAAGTACTCT	CGGACTCATCGTACTCCTGCTT
