# Supplementary material for: SNIP1 and PRC2 coordinate cell fates of neural progenitors during brain development
Source: Nat Commun. 2023 Aug 8;14:4754. doi: 10.1038/s41467-023-40487-4 (PMC10409800; doi:10.1038/s41467-023-40487-4)
Supplement: Supplementary file 3 — Description of Additional Supplementary Files [file 41467_2023_40487_MOESM3_ESM.pdf]

Description of Additional Supplementary Files

File Name: Supplementary Data 1

Description: **<u>Lists of genes with change in SNIP1 enrichment levels</u>**

We enlist genes whose SNIP1 CUT&RUN signals were significantly changed ($p < 0.05$) by inhibitor treatments. The lists for Fig 4j-m have genes that had consistent SNIP1 binding changes between at least two inhibitors. The list for K02288 has genes that had consistent SNIP1 binding changes by K02288 treatment. Two replicates of SNIP1 CUT&RUN were analyzed.
