# Supplementary material for: SNIP1 and PRC2 coordinate cell fates of neural progenitors during brain development
Source: Nat Commun. 2023 Aug 8;14:4754. doi: 10.1038/s41467-023-40487-4 (PMC10409800; doi:10.1038/s41467-023-40487-4)
Supplement: Supplementary file 5 — Reporting Summary [file 41467_2023_40487_MOESM5_ESM.pdf]

nature portfolio

Corresponding author(s): Jamy C Peng

Last updated by author(s): Jul 21, 2023

Reporting Summary

Nature Portfolio wishes to improve the reproducibility of the work that we publish. This form provides structure for consistency and transparency in reporting. For further information on Nature Portfolio policies, see our Editorial Policies and the Editorial Policy Checklist.

Statistics

For all statistical analyses, confirm that the following items are present in the figure legend, table legend, main text, or Methods section.

n/a	Confirmed	
☐	☒	The exact sample size (*n*) for each experimental group/condition, given as a discrete number and unit of measurement
☐	☒	A statement on whether measurements were taken from distinct samples or whether the same sample was measured repeatedly
☐	☒	The statistical test(s) used AND whether they are one- or two-sided<br>*Only common tests should be described solely by name; describe more complex techniques in the Methods section.*
☐	☒	A description of all covariates tested
☐	☒	A description of any assumptions or corrections, such as tests of normality and adjustment for multiple comparisons
☐	☒	A full description of the statistical parameters including central tendency (e.g. means) or other basic estimates (e.g. regression coefficient) AND variation (e.g. standard deviation) or associated estimates of uncertainty (e.g. confidence intervals)
☐	☒	For null hypothesis testing, the test statistic (e.g. *F*, *t*, *r*) with confidence intervals, effect sizes, degrees of freedom and *P* value noted<br>*Give P values as exact values whenever suitable.*
☒	☐	For Bayesian analysis, information on the choice of priors and Markov chain Monte Carlo settings
☐	☒	For hierarchical and complex designs, identification of the appropriate level for tests and full reporting of outcomes
☐	☒	Estimates of effect sizes (e.g. Cohen's *d*, Pearson's *r*), indicating how they were calculated

Our web collection on statistics for biologists contains articles on many of the points above.

Software and code

Policy information about availability of computer code

Data collection

For FACS analysis, data were collected with the software   (Yurika: software name and version #}.
For IF analysis, images were captured on Nikon C2 using NIS-Elements 5.30.05 (Build 1559).
For WB analysis, images were captured on Odyssey® Fc imaging system (LI-COR) using the Image Studio™ software version 1.0.14.

Data analysis

Image analysis is performed on the Image J2 FIJI software, version 2.9.0/1.53t.
Statistical analyses were performed using R version 4.0.1 or Prism version 9.0.2.
Codes for CUT&RUN analyses are avaialable at https://doi.org/10.6084/m9.figshare.7411835.

RNA-seq
Raw reads were first trimmed using TrimGalore (version 0.6.3) available at: https://www.bioinformatics.babraham.ac.uk/projects/trim_galore/, with parameters '--paired --retain_unpaired'. Filtered reads were then mapped to the Mus musculus reference genome (GRCm38.p6 + Gencode-M22 Annotation) using STAR (version 2.7.9a) (Dobin and Gingeras, 2015) [PMID: 26334920]. Gene-level read quantification was done using RSEM (version 1.3.1) (Li and Dewey, 2011). To identify the differentially expressed genes between control and experimental samples, the variation in the library size between samples was first normalized by trimmed mean of M values (TMM) and genes with CPM < 1 in all samples were eliminated. Then, the normalized data were applied to linear modeling with the voom from the limma R package (Law et al., 2014). Gene set enrichment analysis (GSEA) was performed against using the MSigDB database (version 7.1), and differentially expressed genes were ranked based on the their log2(FC) * -log10(p-value) (Liberzon et al., 2015; Subramanian et al., 2005).

CUT&RUN
The reads were aligned to mouse mm10 genome reference and fruit fly dm6 genome reference by BWA (version 0.7.170.7.12, default parameter). Duplicated reads were marked by the bamsormadup from the biobambam tool (version 2.0.87) available at https://

www.sanger.ac.uk/tool/biobambam/. Uniquely mapped reads were kept by samtools (parameter "-q 1 -F 1804," version 1.14). Fragments < 2000 bp were kept for peak calling and bigwig files were generated for visualization. SICER (Xu et al., 2014) and macs2 (Zhang et al., 2008) were both used for peak calling to identify both the narrow and broad peak correctly. With SICER, we assigned peaks that were at the top 1 percentile as the high-confidence peaks and the top 5 percentile as the low-confidence peaks. Two sets of peaks were generated: Strong peaks called with parameter 'FDR < 0.05' by at least one method (macs2 or SICER) and weak peaks called with parameter 'FDR < 0.5' by at least one method (macs2 or SICER). Peaks were considered reproducible if they were supported by a strong peak from all replicates or at least one strong peak and a weak peak in the other replicates. For downstream analyses, heatmaps were generated by deepTools (Ramirez et al., 2014) and gene ontology was performed with Enrichr (Chen et al., 2013; Kuleshov et al., 2016) and GSEA, in addition to custom R scripts. For differential peak analysis, peaks from two replicates were merged and counted for number of overlapping extended reads for each sample (bedtools v2.24.0) (Quinlan and Hall, 2010). Then, we detected the differential peaks by the empirical Bayes method (eBayes function from the limma R package) (Law et al., 2014). Peaks were annotated based on Gencode following this priority: "Promoter.Up": if they fall within TSS - 2kb, "Promoter.Down": if they fall within TSS - 2kb, "exonic" or "intronic": if they fall within an exon or intron of any isoform, "TES peaks": if they fall within TES ⍰ 2kb, "distal5" or "distal3" if they are with 50kb upstream of TSS or 50kb downstream of TES, respectively, and they are classified as "intergenic" if they do not fit in any of the previous categories.

For manuscripts utilizing custom algorithms or software that are central to the research but not yet described in published literature, software must be made available to editors and reviewers. We strongly encourage code deposition in a community repository (e.g. GitHub). See the Nature Portfolio guidelines for submitting code & software for further information.

Data

Policy information about availability of data

All manuscripts must include a data availability statement. This statement should provide the following information, where applicable:

- Accession codes, unique identifiers, or web links for publicly available datasets
- A description of any restrictions on data availability
- For clinical datasets or third party data, please ensure that the statement adheres to our policy

All sequencing data are deposited in GEO: GSE212445. Reads were mapped to the Mus musculus reference genome (GRCm38.p6 + Gencode-M22 Annotation).

Human research participants

Policy information about studies involving human research participants and Sex and Gender in Research.

Reporting on sex and gender	N/A.
Population characteristics	N/A.
Recruitment	N/A.
Ethics oversight	N/A.

Note that full information on the approval of the study protocol must also be provided in the manuscript.

Field-specific reporting

Please select the one below that is the best fit for your research. If you are not sure, read the appropriate sections before making your selection.

☒ Life sciences ☐ Behavioural & social sciences ☐ Ecological, evolutionary & environmental sciences

For a reference copy of the document with all sections, see nature.com/documents/nr-reporting-summary-flat.pdf

Life sciences study design

All studies must disclose on these points even when the disclosure is negative.

Sample size	The sample size were determined by a statistical power analysis using the online tool, powerandsamplesize.com/Calculators. Most calculations were done with the intention to compare 2 means, with 2-sample, 1-sided assumption.
Data exclusions	No exclusion.
Replication	Observations were repeated at least 2 times. RNA-seq was done with wildtype, Snip1 conditional KO, and Snip1-Eed conditional KO cells with at least 3 replicates. Chromatin profiling was done with CUT&RUN with wildtype vs. Snip1-conditional knockout mouse NPCs with mostly 2 replicates. Replication were successful.
Randomization	We did not perform randomization, which is not relevant to this study.<br>Experimental groups were wildtype, Snip1 conditional KO, and Snip1-Eed conditional KO embryos. The groups were analyzed by pair-wise analyses to address specific hypotheses. Because of the nature of addressing these hypotheses (biological questions), randomization is not applicable.

Blinding	During data collection, investigators could not be blinded to group allocation. Blinding was not possible because the experimental groups (wild-type, Snip1 conditional KO, and Snip1-Eed conditional KO embryos) were structurally distinct from each other. Snip1 conditional KO embryos had severe brain tissue atrophy, which were quite structurally distinct from wild-type and also different from Snip1-Eed conditional KO. At sample collection and onward, experimentalists saw the structurally variant samples and could not be blinded.<br>Data analysis was not blinded. However, seq data were analyzed without assumption of data trend, 'unsupervised.'

Reporting for specific materials, systems and methods

We require information from authors about some types of materials, experimental systems and methods used in many studies. Here, indicate whether each material, system or method listed is relevant to your study. If you are not sure if a list item applies to your research, read the appropriate section before selecting a response.

Materials & experimental systems

n/a	Involved in the study
☐	☒ Antibodies
☐	☒ Eukaryotic cell lines
☒	☐ Palaeontology and archaeology
☐	☒ Animals and other organisms
☒	☐ Clinical data
☒	☐ Dual use research of concern

Methods

n/a	Involved in the study
☐	☒ ChIP-seq
☐	☒ Flow cytometry
☒	☐ MRI-based neuroimaging

Antibodies

Antibodies used	Antibody, Species, Source, Catalogue Number, Dilution<br>Anti-Normal IgG, rabbit, RD Systems, AB-105-C, IP (ug) CUT&RUN (1ug)<br>Anti-Normal IgG, goat, RD Systems, AB-108-C, IP (ug)<br>Anti-Jarid2, Novus Biological, NB100-2214, IP (ug) WB (1:1000)<br>Anti-Ezh2, Active Motif, 39934, WB (1:1000)<br>Anti-Ezh2, Active Motif, 39076, IP (ug) WB (1:1000) CUT&RUN (1ug)<br>Anti-Suz12, Cell Signaling Technology, 3737, WB (1:1000) CUT&RUN (1ug)<br>Anti-Suz12, Active Motif, 39057, WB (1:1000) CUT&RUN (1ug)<br>Anti-Snip1, ThermoFisher, 29412, IP(ug) IF (1:50) WB (1:1000) CUT&RUN (1ug)<br>Anti-Snip1, ProteinTech, 14950-I-AP, IP (ug) WB (1:1000)<br>Anti-Rbbp5, Bethyl Laboratories, A300-109A, WB (1:1000)<br>Anti-p300, RD Systems, AF3789, IP (ug) WB (1:1000)<br>Anti-Cbp, GeneTex, GTX101249, IP (ug) WB (1:1000)<br>Anti-Drosophila H2Av, Active Motif, 61686, CUT&RUN (0.25ug)<br>Anti-p53, Leica Biosystems, NCL-L-p53-CM5p, IF (1:100)<br>Anti-Histone H3, Rockland 100-401-E81, WB (1:2000)<br>Anti-H3K27me3, Millipore, 07-449, CUT&RUN (0.5ug)<br>Anti-H3K27ac, Abcam, ab4729, CUT&RUN (0.5ug)<br>Anti-γH2AX, Cell Signaling Technology, 9718, IF (1:100)<br>Anti-γH2AX, Millipore, 05-636, WB (1:1000)<br>Anti-β-Actin, Sigma-Aldrich, A1978, WB (1:2000)<br>Anti-Sox2, Santa Cruz Biotechnology, sc-17320, IF (1:150)<br>Anti-Tbr2, ThermoFisher, 14-4875-80, IF (1:200)<br>Anti-Insm1-Alexa Fluor® 488, Santa Cruz Biotechnology, sc-271408 AF488, IF (1:50)<br>Anti-Tuj1, Sigma-Aldrich, T8660, IF (1:300)<br>Anti-Map2, Abcam, ab5392, IF (1:5000)<br>Anti-cleaved caspase 3 (Asp175), Cell Signaling Technology, 9661, IF (1:200)<br>Anti-cleaved caspase 8 (Asp387), Cell Signaling Technology, 8592, IF (1:200)<br>Anti-cleaved caspase 9 (Asp353), Cell Signaling Technology, 9509, IF (1:200)<br>Anti-Ki-67 Cell Signaling Technology 9129 IF (1:100)<br>Anti-BrdU, Santa Cruz Biotechnology, 32323, IF (1:100)<br>Anti-Foxg1, Abcam, ab196868, IF (1:200)<br>Anti-Otx2, R&D Systems, AF1979, IF (1:150)<br>IRDye®-conjugated secondary antibodies, LI-COR, 926-32213, 926-68072, WB (1:17,000)<br>Clean-Blot™ IP detection reagent, ThermoFisher, 21230, WB (1:3000)
Validation	All antibodies were purchased from commercial vendors based on previous validation from publications from highly reputable labs/ sources/ENCODE project. For most antibodies, western blotting and immunofluorescence were performed to ensure that the antibody recognizes 1 protein at the right size or immunofluorescence signals localize to the right structures in the cells. For Snip1 antibodies, protein depletion was used in western blotting for validation. More detailed validation information is listed below.<br>Anti-Jarid2, murine knockout validated by Novus Biological (https://www.novusbio.com/products/jumonji-jarid2-antibody_nb100-2214)<br>Anti-Ezh2, murine reactivity tested by Active Motif (https://www.activemotif.com/catalog/details/39933/ezh2-antibody-pab)<br>Anti-Ezh2, murine reactivity tested by Active Motif (https://www.activemotif.com/catalog/details/39875/ezh2-antibody-mab-clone-ac22)

Anti-Suz12, murine reactivity tested by Cell Signaling Technology (https://www.cellsignal.com/products/primary-antibodies/suz12-d39f6-xp-rabbit-mab/3737)
Anti-Suz12, murine reactivity tested by Active Motif (https://www.activemotif.com/catalog/details/39357/suz12-antibody-pab)
Anti-Snip1, validated by our murine knockout samples (https://www.thermofisher.com/antibody/product/SNIP1-Antibody-Polyclonal/PA5-29412)
Anti-Snip1, validated by our murine knockout samples (https://www.ptglab.com/products/SNIP1-Antibody-14950-1-AP.htm)
Anti-Rbbp5, murine reactivity tested by Bethyl Laboratories (https://www.thermofisher.com/antibody/product/RbBP5-Antibody-Polyclonal/A300-109A-T)
Anti-p300, (https://www.rndsystems.com/products/human-p300-antibody_af3789)
Anti-Cbp, human knockdown/out validated by GeneTex (https://www.genetex.com/Product/Detail/CBP-antibody-C3-C-term/GTX101249)
Anti-Drosophila H2Av, ChIP validated by Active Motif (https://www.activemotif.com/catalog/1091/chip-normalization)
Anti-p53, murine reactivity tested by Leica Biosystems (https://shop.leicabiosystems.com/us/ihc-ish/ihc-primary-antibodies/pid-p53-protein-cm5)
Anti-Histone H3, (https://www.rockland.com/categories/primary-antibodies/histone-h3-antibody-100-401-E81/)
Anti-H3K27me3, murine reactivity tested by Millipore (https://www.emdmillipore.com/US/en/product/Anti-trimethyl-Histone-H3-Lys27-Antibody,MM_NF-07-449)
Anti-H3K27ac, murine reactivity tested by Abcam (https://www.abcam.com/products/primary-antibodies/histone-h3-acetyl-k27-antibody-chip-grade-ab4729.html)
Anti-γH2AX, murine reactivity tested by Cell Signaling Technology (https://www.cellsignal.com/products/primary-antibodies/phospho-histone-h2a-x-ser139-20e3-rabbit-mab/9718)
Anti-γH2AX (https://www.emdmillipore.com/US/en/product/Anti-phospho-Histone-H2A.X-Ser139-Antibody-clone-JBW301,MM_NF-05-636)
Anti-β-Actin, murine reactivity tested by Millipore-Sigma (https://www.sigmaaldrich.com/US/en/product/sigma/a1978)
Anti-Sox2, (https://www.scbt.com/p/sox-2-antibody-y-17)
Anti-Tbr2, murine reactivity tested by ThermoFisher (https://www.thermofisher.com/antibody/product/EOMES-Antibody-clone-Dan11mag-Monoclonal/14-4875-82)
Anti-Insm1-Alexa Fluor® 488, murine reactivity tested by Santa Cruz Biotechnology (https://www.scbt.com/p/insm1-antibody-a-8)
Anti-Tuj1, murine reactivity tested by Sigma-Aldrich (https://www.sigmaaldrich.com/US/en/product/sigma/zrb1140?gclid=Cj0KCQjwk96lBhDHARIsAEKO4xYocFXvlunCYu1BNeA_ZweQ6oeoMXKTCpk7at2eDDrZDihFZSPKCXkaAr2cEALw_wcB&gclsrc=aw.ds)
Anti-Map2, murine reactivity tested by Abcam (https://www.abcam.com/products/primary-antibodies/map2-antibody-ab5392.html)
Anti-cleaved caspase 3 (Asp175), murine reactivity tested by Cell Signaling Technology (https://www.cellsignal.com/products/primary-antibodies/cleaved-caspase-3-asp175-antibody/9661)
Anti-cleaved caspase 8 (Asp387), murine reactivity tested by Cell Signaling Technology (https://www.cellsignal.com/products/primary-antibodies/cleaved-caspase-8-asp387-d5b2-xp-rabbit-mab/8592)
Anti-cleaved caspase 9 (Asp353), murine reactivity tested by Cell Signaling Technology (https://www.cellsignal.com/products/primary-antibodies/cleaved-caspase-9-asp353-antibody/9509?_=1689802082279&Ntt=9509&tahead=true)
Anti-Ki-67, murine reactivity tested by Cell Signaling Technology (https://www.cellsignal.com/products/primary-antibodies/ki-67-d3b5-rabbit-mab/9129)
Anti-BrdU, (https://www.scbt.com/p/brdu-antibody-iib5)
Anti-Foxg1, murine reactivity tested by Abcam (https://www.abcam.com/products/primary-antibodies/foxg1-antibody-epr18987-ab196868.html)
Anti-Otx2, murine reactivity tested by R&D Systems (https://www.rndsystems.com/products/human-otx2-antibody_af1979?gclid=Cj0KCQjwk96lBhDHARIsAEKO4xb4-f4G96OjftdTaPd0zZ5uIqkYXl-Lzs9IKEokv_EJnqgb8XSrsSMaAsu9EALw_wcB&gclsrc=aw.ds)

Eukaryotic cell lines

Policy information about cell lines and Sex and Gender in Research

Cell line source(s)	Drosophila S2 cells (catalog #6) were purchased from Drosophila Genomics Resource Center. Cells were used as spike-in for normalization between CUT&RUN replicates.
Authentication	Cells were authenticated by the commercial source and deep-sequenced.
Mycoplasma contamination	Cells were tested negative for mycoplasma.
Commonly misidentified lines (See ICLAC register)	N/A.

Animals and other research organisms

Policy information about studies involving animals; ARRIVE guidelines recommended for reporting animal research, and Sex and Gender in Research

Laboratory animals

Strain, Genotype, Source, Description
Snip1-tm1a, B6Dnk;B6N-Snip1<tm1a(EUCOMM)Wtsi>/H, Infrafrontier/EMMA 04224
Eed-flox, B6;129S1-Eedtm1Sho/J, JAX Stock 022727, Yu et al., 2009
Actin-FLPe, B6;SJL-Tg(ACTFLPe)9205Dym/J, a gift from Dr. Peter McKinnon at St. Jude Children's Research Hospital/JAX Stock 003800, Rodriguez et al., 2000
Nestin-Cre, B6.Cg-Tg(Nes-Cre)1Kln/J, JAX Stock 003771, Tronche et al., 1999
Emx1-Cre, B6.129S2-Emx1tm1(cre)Krj/J, a gift from Dr. Peter McKinnon at St. Jude Children's Research Hospital/JAX Stock 005628, Lee et al., 2012
Sox2-eGFP, B6;129S1-Sox2tm1Hoch/J, JAX Stock 017592, Arnold et al., 2011

	Animals at age 2-12 months were used for timed mating crosses to yield embryos at E11.5-13.5 for analyses.
Wild animals	None.
Reporting on sex	Male and female mouse embryos were randomly assigned in this study. Because our genes of interest are not located on any of the sex chromosomes, phenotypes and/or molecular observations are expected to be similar between males and females in our study.
Field-collected samples	None.
Ethics oversight	IACUC at St. Jude approved and oversaw the breeding and use of the mouse animals in this study.

Note that full information on the approval of the study protocol must also be provided in the manuscript.

ChIP-seq

Data deposition

☒ Confirm that both raw and final processed data have been deposited in a public database such as GEO.

☒ Confirm that you have deposited or provided access to graph files (e.g. BED files) for the called peaks.

Data access links
May remain private before publication.

https://www.ncbi.nlm.nih.gov/geo/query/acc.cgi?acc=GSE212444

Files in database submission

CUT&RUN
GSM6532897 E13_brainGFPpos_SNC1132-1_Snip1CKO_H3K27ac
GSM6532898 E13_brainGFPpos_SNC1132-2_Snip1WT_H3K27ac
GSM6532899 E13_brainGFPpos_SNC1134-1_Snip1CKO_H3K27ac
GSM6532900 E13_brainGFPpos_SNC1134-2_Snip1WT_H3K27ac
GSM7233861 E13_brainGFPpos_SNC42-7_Snip1WT_H3K27ac
GSM7233862 E13_brainGFPpos_SNC42-4_Snip1CKO_H3K27ac
GSM6532901 E13_brainGFPpos_SNC1250-1_Snip1CKO_Snip1TH
GSM6532902 E13_brainGFPpos_SNC1250-2_Snip1WT_Snip1TH
GSM6532903 E13_brainGFPpos_SNC1250-3_Snip1WT_Snip1TH
GSM6532908 E13_brainGFPpos_SNC72-6_Snip1CKO_Snip1TH
GSM6532904 E13_brainGFPpos_SNC176-2_Snip1CKO_H3K27me3
GSM6532905 E13_brainGFPpos_SNC176-3_Snip1CKO_H3K27me3
GSM6532906 E13_brainGFPpos_SNC176-4_Snip1WT_H3K27me3
GSM6532907 E13_brainGFPpos_SNC176-6_Snip1WT_H3K27me3
GSM6532909 E13_Sox2GFP_Ctrl_1374_1_Ezh2
GSM6532912 E13_Sox2GFP_Snip1CKO_178_5_Ezh2
GSM6532910 E13_Sox2GFP_Ctrl_1374_1_Suz12AM
GSM6532911 E13_Sox2GFP_Ctrl_1374_2_Suz12AM
GSM6532913 E13_Sox2GFP_Snip1CKO_2830_1_Suz12AM
GSM6532914 E13_Sox2GFP_Snip1CKO_2830_2_Suz12AM

CUT&RUN with inhibitors
GSM7221135 culturedmNPCs_BAY11_7082_Snip1TH_rep2
GSM7221136 culturedmNPCs_K02288_Snip1TH_rep2
GSM7221137 culturedmNPCs_DMSO_Snip1TH_rep1
GSM7221138 culturedmNPCs_JSH23_Snip1TH_rep2
GSM7221139 culturedmNPCs_Galunisertib_Snip1TH_rep2
GSM7221140 culturedmNPCs_DMSO_IgG_rep1
GSM7221141 culturedmNPCs_K02288_Snip1TH_rep1
GSM7221142 culturedmNPCs_LDN193189_Snip1TH_rep2
GSM7221143 culturedmNPCs_BAY11_7082_Snip1TH_rep1
GSM7221144 culturedmNPCs_LDN193189_Snip1TH_rep1
GSM7221145 culturedmNPCs_DMSO_Snip1TH_rep2
GSM7221146 culturedmNPCs_Galunisertib_Snip1TH_rep1
GSM7221147 culturedmNPCs_JSH23_Snip1TH_rep1
GSM7221148 culturedmNPCs_DMSO_IgG_rep2
GSM7221149 culturedmNPCs_IKK16_Snip1TH_rep2
GSM7221150 culturedmNPCs_IKK16_Snip1TH_rep1

CUT&RUN-reChIP
GSM7221151 Snip1TH_IP_Suz12_rep2
GSM7221152 Snip1TH_IP_H3K27me3_rep2
GSM7221153 Snip1TH_IP_H3K27me3_rep1
GSM7221154 Snip1TH_IP_Ezh2_rep1
GSM7221155 Snip1TH_IP_IgG_rep1
GSM7221156 Snip1TH_IP_Ezh2_rep2
GSM7221157 Snip1TH_IP_IgG_rep2
GSM7221158 Snip1TH_IP_Suz12_rep1

CUT&RUN with lentiviral Cre transduction
GSM7221159 Day2_Cre_Ezh2_rep1
GSM7221160 Day2_Cre_Suz12_rep2
GSM7221161 Day2_Empty_Suz12_rep2
GSM7221162 Day3_Cre_Suz12_rep2
GSM7221163 Day3_Cre_Ezh2_rep1
GSM7221164 Day2_Empty_Ezh2_rep1
GSM7221165 Day3_Empty_Ezh2_rep1
GSM7221166 Day2_Empty_Suz12_rep1
GSM7221167 Day2_Empty_Ezh2_rep2
GSM7221168 Day3_Cre_Ezh2_rep2
GSM7221169 Day3_Empty_Suz12_rep2
GSM7221170 Day2_Cre_Ezh2_rep2
GSM7221171 Day3_Empty_Ezh2_rep2
GSM7221172 Day2_Cre_Suz12_rep1
GSM7221173 Day3_Empty_Suz12_rep1
GSM7221174 Day3_Cre_Suz12_rep1

Genome browser session
(e.g. UCSC)

none.

Methodology

Replicates

CUT&RUN
Snip1 CUT&RUN: 2 replicates
H3K27me3 CUT&RUN: 2 replicates
H3K27ac CUT&RUN: 3 replicates
Suz12 CUT&RUN: 2 replicates
Ezh2 CUT&RUN: 1 replicate for wildtype and Snip1 conditional KO each

CUT&RUN with inhibitors
IgG CUT&RUN: 2 replicates per inhibitor
Snip1 CUT&RUN: 2 replicates per inhibitor

CUT&RUN-reChIP
IgG CUT&RUN: 2 replicates
H3K27me3 CUT&RUN: 2 replicates
Suz12 CUT&RUN: 2 replicates
Ezh2 CUT&RUN: 2 replicates

CUT&RUN with lentiviral Cre transduction
Suz12 CUT&RUN: 2 replicates
Ezh2 CUT&RUN: 2 replicates

Sequencing depth

All the CUT&RUN samples had > 5 million uniquely mapped reads as recommended.

Antibodies

Antibody, Species, Source, Catalogue Number
Anti-Normal IgG, rabbit, RD Systems, AB-105-C
Anti-Ezh2, Active Motif, 39076
Anti-Suz12, Cell Signaling Technology, 3737
Anti-Suz12, Active Motif, 39057
Anti-Snip1, ThermoFisher, 29412
Anti-Drosophila H2Av, Active Motif, 61686
Anti-H3K27me3, Millipore, 07-449
Anti-H3K27ac, Abcam, ab4729

Peak calling parameters

With fragments < 2000 bp, SICER (Xu et al., 2014) and macs2 (Zhang et al., 2008) were both used for peak calling to identify both the narrow and broad peak correctly. With SICER, we assigned peaks that were at the top 1 percentile as the high-confidence peaks and the top 5 percentile as the low-confidence peaks. Two sets of peaks were generated: Strong peaks called with parameter 'FDR < 0.05' by at least one method (macs2 or SICER) and weak peaks called with parameter 'FDR < 0.5' by at least one method (macs2 or SICER). Peaks were considered reproducible if they were supported by a strong peak from all replicates or at least one strong peak and a weak peak in the other replicates.

Data quality

We visualized CUT&RUN peaks on the Integrated Genomics Viewer (Broad Institute) to validate called peaks and the consistency among replicates. We also calculated the Pearson correlation coefficient among replicates, which suggested high reproducibility.

Software

The reads were aligned to mouse mm10 genome reference and fruit fly dm6 genome reference by BWA (version 0.7.170.7.12, default parameter). Duplicated reads were marked by the bamsormadup from the biobambam tool (version 2.0.87) available at https://www.sanger.ac.uk/tool/biobambam/. Uniquely mapped reads were kept by samtools (parameter "-q 1 -F 1804," version 1.14). Fragments < 2000 bp were kept for peak calling and bigwig files were generated for visualization. SICER (Xu et al., 2014) and macs2 (Zhang et al., 2008) were both used for peak calling to identify both the narrow and broad peak correctly. With SICER, we assigned peaks that were at the top 1 percentile as the high-confidence peaks and the top 5 percentile as the low-confidence peaks. Two sets of peaks were generated: Strong peaks called with parameter 'FDR < 0.05' by at least one method (macs2 or SICER) and weak peaks called with parameter 'FDR < 0.5' by at least one method (macs2 or SICER). Peaks were considered reproducible if they were supported by a strong peak from all replicates or at least one strong peak and a weak peak in the other replicates. For

downstream analyses, heatmaps were generated by deepTools (Ramirez et al., 2014) and gene ontology was performed with Enrichr (Chen et al., 2013; Kuleshov et al., 2016) and GSEA, in addition to custom R scripts. For differential peak analysis, peaks from two replicates were merged and counted for number of overlapping extended reads for each sample (bedtools v2.24.0) (Quinlan and Hall, 2010). Then, we detected the differential peaks by the empirical Bayes method (eBayes function from the limma R package) (Law et al., 2014). Peaks were annotated based on Gencode following this priority: “Promoter.Up”: if they fall within TSS - 2kb, “Promoter.Down”: if they fall within TSS - 2kb, “exonic” or “intronic”: if they fall within an exon or intron of any isoform, “TES peaks”: if they fall within TES ⍰ 2kb, “distal5” or “distal3” if they are with 50kb upstream of TSS or 50kb downstream of TES, respectively, and they are classified as “intergenic” if they do not fit in any of the previous categories.

Flow Cytometry

Plots

Confirm that:

☒ The axis labels state the marker and fluorochrome used (e.g. CD4-FITC).

☒ The axis scales are clearly visible. Include numbers along axes only for bottom left plot of group (a 'group' is an analysis of identical markers).

☒ All plots are contour plots with outliers or pseudocolor plots.

☒ A numerical value for number of cells or percentage (with statistics) is provided.

Methodology

Sample preparation	The Sox2-eGFP embryos were dissected out from the uterus and visceral yolk sac at embryonic day 13.5 (E13.5). Brains were dissected from embryos under the dissection microscope in cold 1x PBS. 300 μL Dulbecco’s Modified Eagle’s Medium (DMEM) (ATCC 30-2002) and 150 μL of 10 mg/mL collagenase Type II (Worthington LS004176) were added to each brain and incubated for 5-10 min at 37 °C. After centrifugation at 1000 xg for 3 min, the tissue was incubated with 500 μL 0.25% Trypsin-EDTA (ThermoFisher 25200056) for 5 min at 37 °C. Trypsinization was quenched with 500 μL DMEM supplemented with 10% fetal bovine serum (FBS) and pelleted by centrifugation at 1000 xg for 3 min. Alternatively, cells were dissociated from the brain using the papain dissociation system (Worthington LK003153). Sox2-eGFP-positive neural progenitor cells (NPCs) were collected by fluorescence-activated cell sorting (FACS).<br><br>FACS-based cell death assay<br>5 X 10^5 NPCs from Snip1[+/+] and Snip1[flox/flox] embryos were seeded onto each well of matrigel-coated 6-well plates. On the following day, cells were incubated with mCherry-Cre lentivirus (Vector Core Lab at St. Jude Children’s Research Hospital) for 8 hours, washed twice with 1X PBS, and cultured for 3 days. To quantify the population of cells with active caspases 3 and 7, cells were incubated at 37 °C with reconstituted FAM-FLICA® at a 1:300 dilution (ImmunoChemistry Technologies 94) for 30 min. Cells were fixed in a 4% formaldehyde solution at room temperature for 15 min and washed twice with 1X PBS. FAM-FLICA–positive cells were quantified by FACS (Excitation: 492 nm, Emission: 520 nm). FACS data were analyzed by FlowJo. To examine whether cell death is via activation of caspase 8 or 9, Z-IETD-FMK (a caspase 8 inhibitor) and Z-LEHD-FMK TFA (a caspase 9 inhibitor) were dissolved in DMSO at 50mM (Compound Management Center at St. Jude Children’s Research Hospital). After cells were incubated with mCherry-Cre lentivirus for 8 hours, these compounds were added at a series of concentrations and incubated for 3 days before FACS analysis. For all the inhibitor treatment assays, medium with the inhibitors was changed every 2 days.
Instrument	S3e Cell Sorter
Software	ProSort software
Cell population abundance	About 2-5 million cells were used in each sort. The GFP-positive fraction was about 80-90% of populations depending on the genotype.<br><br>FACS-based cell death assay<br>More than 1 X10^4 cells were analyzed in each sample. The FAM-FLICA–positive fraction ranged from close to 0% to around 15%, depending on the genotype and presence/absence of Cre recombinase.
Gating strategy	Primary gates are the 'live cells' and the 'single cells' gates. Positive signal gate is set to distinguish negative from positive events. Dim signals are eliminated from the gate to ensure positives. Events from the gate are back-gated to the primary gates to ensure events are well within the bounds of the initial gates.

☒ Tick this box to confirm that a figure exemplifying the gating strategy is provided in the Supplementary Information.
